# The effects of playbacks of anthropogenic noise on the anti‐predator behaviour of Pacific sand lance, *Ammodytes personatus*

**DOI:** 10.1111/jfb.70642

**Published:** 2026-09-07

**Authors:** Meredith A. V. White, Francis Juanes, Patrick D. O'Hara, Jose Tavera, Matthew R. Baker, Douglas F. Bertram, Adam Summers, David A. Fifield, Nora V. Carlson

**Affiliations:** ^1^ Department of Biology University of Victoria Victoria British Columbia Canada; ^2^ Institute of Ocean Sciences Environment and Climate Change Canada, Integrated Marine Spatial Ecology Lab Sidney British Columbia Canada; ^3^ Universidad del Valle Santiago de Cali Colombia; ^4^ School of Aquatic and Fishery Sciences University of Washington Seattle Washington USA; ^5^ Friday Harbor Laboratories University of Washington Friday Harbor Washington USA; ^6^ Wildlife Research Division, Science and Technology Branch, Environment and Climate Change Canada Mount Pearl Newfoundland and Labrador Canada

**Keywords:** anthropogenic noise, disturbance, forage fish, predator–prey relationships, stress

## Abstract

Noise from human activities has increased and been shown to negatively impact marine animals. Marine mammals and fish are vulnerable to anthropogenic noise because they rely on sound to communicate, find food, avoid predators and sense their environment. Forage fish are small schooling pelagic fish, important to many predators and instrumental in moving energy to higher trophic levels. There are limited studies investigating how noise influences forage fish behaviour, especially their anti‐predator behaviour. Here, we investigate how noise from boats, pile driving and wind farms affects the anti‐predator behaviour of Pacific sand lance (*Ammodytes personatus*, PSL), a key forage fish in the Northeast Pacific Ocean. We exposed PSL to four different noise playbacks and documented anti‐predator behaviour (i.e., burying in the sand and startling). We hypothesized that exposure to anthropogenic noise would increase anti‐predator behaviour in PSL. Contrary to our hypothesis, exposure to boat noise and pile driving noise decreased startling behaviour and burying behaviour, while wind farm noise increased both. These results suggest that different noise sources may impact behaviour through different mechanisms such as noise‐induced stress or distraction and, as a consequence, can both increase PSL short‐ term availability in some contexts while decreasing availability overall. Understanding how fish and their predators respond to different noise sources over longer periods of time is critical in understanding the effects of noise on marine communities and necessary to facilitate recommendations on the management of marine anthropogenic stressors and highlight conservation efforts for coastal areas.

## 1 INTRODUCTION

The acoustic environment has changed in terrestrial and aquatic habitats around the world due to the steep rise in human‐generated or anthropogenic noise since the industrial revolution (Frisk, 2012; McDonald et al., 2006; Simmonds et al., 2014). Human activities including natural resource exploitation, ground and air traffic, urbanization and shipping cause harmful effects on animals such as fish, invertebrates, birds and marine mammals (Bayne et al., 2008; Dahlheim & Castellote, 2016; Halvorsen et al., 2012; Schmidt et al., 2014). Anthropogenic noise is considered a pollutant of international concern, recognized in legislation in Europe and parts of North and South America (European Union, 2008; National Environmental Policy Act of 1969, 1970; Colombian law 2450 of 2025), and is of particular concern in marine habitats as sound propagates faster underwater (Slabbekoorn et al., 2010). Marine anthropogenic noise is highly concentrated in coastal regions, which are experiencing human population increases and concomitant increases in commercial shipping and recreational boating (Davenport & Davenport, 2006; Small & Nicholls, 2003). Vessel noise is the main source of anthropogenic noise in coastal regions, producing low‐frequency sounds that fall within the vocalization and hearing ranges of many marine organisms (Duarte et al., 2021). However, growing demand for offshore wind farms (OWFs) and the associated construction (pile driving) and operation of these farms can also alter the acoustic environment, though these noise impacts on marine life are poorly understood (Gill et al., 2020; van der Knaap, Slabbekoorn, et al., 2022).

Anthropogenic noise poses a threat to fish because it can cause physiological, behavioural and physical effects (Ivanova et al., 2020; Kunc et al., 2016; Whitfield & Becker, 2014). Anthropogenic noise has been shown to disrupt fish communication (Codarin et al., 2009; Picciulin et al., 2012; Sebastianutto et al., 2011; Wahlberg & Westerberg, 2005), reduce parental care and breeding behaviour (Bruintjes & Radford, 2013; Picciulin et al., 2010), increase stress levels (Bruintjes et al., 2016; Smith et al., 2004; Wysocki et al., 2006), increase anti‐predator behaviour (Hawkins et al., 2014; La Manna et al., 2016; Sarà et al., 2007; van der Knaap, Ashe, et al., 2022), decrease foraging efficiency (Purser & Radford, 2011), negatively impact territorial behaviour (Sebastianutto et al., 2011) and induce behavioural changes in freshwater, estuarine and marine fish (Cox et al., 2018). While research has begun to explore the effects of noise on fish, forage fish have been largely overlooked.

Forage fish are abundant small pelagic schooling fish that facilitate energy transfer between lower and higher trophic levels and make up an important part of the diet of many species of predators (Cury et al., 2000; Springer & Speckman, 1997; von Biela et al., 2019). Many predators of forage fish, including fishes (Engelhard et al., 2008, 2013; Gunther et al., 2024; Rindorf et al., 2008), seabirds (Bertram & Kaiser, 1993; Pikitch et al., 2004; Szoboszlai et al., 2015) and marine mammals (Huag & Nilssen, 1995; MacLeod et al., 2007; Trites, 1992) rely on them as an important resource for their growth and condition. Additionally, forage fish availability impacts seabird reproductive success (Borstad et al., 2011; Carroll et al., 2017; Cury et al., 2011; Hedd et al., 2006; Wanless et al., 1998). While little is known about how forage fish behaviour is influenced by prevalent anthropogenic noise sources (but see Brehmer et al., 2003; Goetz et al., 2015; Jørgensen et al., 2004; Kastelein et al., 2007; Kraus et al., 1997), there is evidence that noise has large negative impacts. Semi‐captive herring (i.e., *Clupea harengus* and *C. pallasii*), for example, have been shown to increase startle and avoidance reactions to passing boats, possibly increasing their vulnerability to predators (Doksæter et al., 2012; van der Knaap, Ashe, et al., 2022). Noise pollution occurring near eulachon (*Thaleichthys pacificus*) spawning areas impact spawning behaviour, possibly reducing abundance (Schweigert et al., 2012). These types of declines in forage fish availability could disrupt energy transfer to higher trophic levels in marine ecosystems (Rovellini et al., 2025; von Biela et al., 2019). As anthropogenic noise levels continue to rise in the Northeast Pacific Ocean (Hildebrand, 2009), concern grows about how forage fish will respond to such noise and the implications for their predators (Schwarz & Greer, 1984; van der Knaap, Ashe, et al., 2022), commercial ventures and to the ecosystem as a whole.

In this study, we investigated the effects of different types of anthropogenic noise on the anti‐predator behaviour of Pacific sand lance (*Ammodytes personatus*, PSL), a zooplanktivorous forage fish important to the Northeast Pacific marine food web. To determine how different noise sources affect PSL behaviour, we brought wild PSL into the laboratory and placed them in one of four different acoustic environments. We observed PSL responses to noise, created an ethogram and compared burying and startling behaviour across noise treatments. Based on the risk‐disturbance hypothesis (Frid & Dill, 2002), which suggests that animals evoke a similar response to both predation and nonlethal human disturbance, we predicted that the fish would respond to anthropogenic noise playbacks in the same manner as a predatory threat, resulting in increases in startling and burying behaviour (Bejder et al., 2009; La Manna et al., 2016; Spiga et al., 2017).

## 2 METHODS

All methods regarding the catching, housing of and conducting playbacks to PSL are the same as those in Carlson et al. (2025).

### 2.1 Study species

PSL is found from Alaska to northern California and is present in the diet of many marine birds, mammals and fish (Robards et al., 1999). PSL are considered hearing non‐specialists (Popper et al., 2022; Popper & Fay, 2011) because they do not possess a swim bladder but can detect sound using their lateral line and otoliths (Robards et al., 1999). There is a lack of information on how noise affects PSL behaviour, but auditory evoked potentials in the American sand lance (*A. americanus*) and the Western sand lance (*A. japonicus*) suggest that their hearing ranges overlap with the frequency ranges of operational windfarms, pile driving noise and vessel noise (Duarte et al., 2021; Strobel & Mooney, 2012; Suga et al., 2005). Sand lance employ three main behaviours to avoid predation: burying in the sand (Robards et al., 1999), startling and schooling (Meyer et al., 1979).

### 2.2 Fish collection and containment

We collected PSL at Jackson Beach (48°31′11.6″ N 123°00′40.3″ W) (Baker et al., 2019, 2023) and Eagle Cove (48°27′41.9″ N 123°01′56.9″ W), San Juan Island, Washington, USA, from 27 April to 10 May 2023. We used a seine net (36.6 m wide × 3.7 m deep; 2.5–4 mm mesh) which we deployed from a boat ~25 m from shore at Jackson Beach, as well as a hand seine (8.6 m wide × 2.1 m deep, 2.5–4 mm mesh) pulled through ~1.5 m deep waters by two people on each end while a third person walked in front of the path of the net to herd schools of fish at Eagle Cove. Nets were pulled completely onto shore, and fish were removed by hand and placed into coolers containing sea water and pumps with air stones. We transported a total of 345 fish to the University of Washington's Friday Harbor Laboratories (FHL), where we randomly assigned individual fish to water‐permeable circular enclosures (0.9 m high × 0.8 m diameter) that were housed within a concrete holding tank (i.e., 1 school/enclosure and 2 enclosures/tank; 8 schools across 4 tanks; Figure 1). We added ~40–50 PSL to each enclosure (treatment = enclosure 1 and enclosure 2 school size; control = 59 and 45 fish; boat noise = 43 and 42 fish; wind farm = 41 and 43 fish; pile driving = 45 and 43 fish). There was a similar mix of age 1 (60–80 mm) and age 2+ (>80 mm; Matta & Baker, 2020) fish in each enclosure (proportion of 2+ fish in a school ± SE calculated from the 30% euthanized measured at the end of the experiment (for details see Carlson et al., 2025); control: 0.44 ± 0.12, boat noise: 0.60 ± 0.07, pile driving noise: 0.41 ± 0.03, wind farm noise: 0.48 ± 0.08). To ensure that the fish were transported from the nets to coolers as fast and as with little handling as possible, we did not take initial length measurements or take pictures.

**FIGURE 1:**
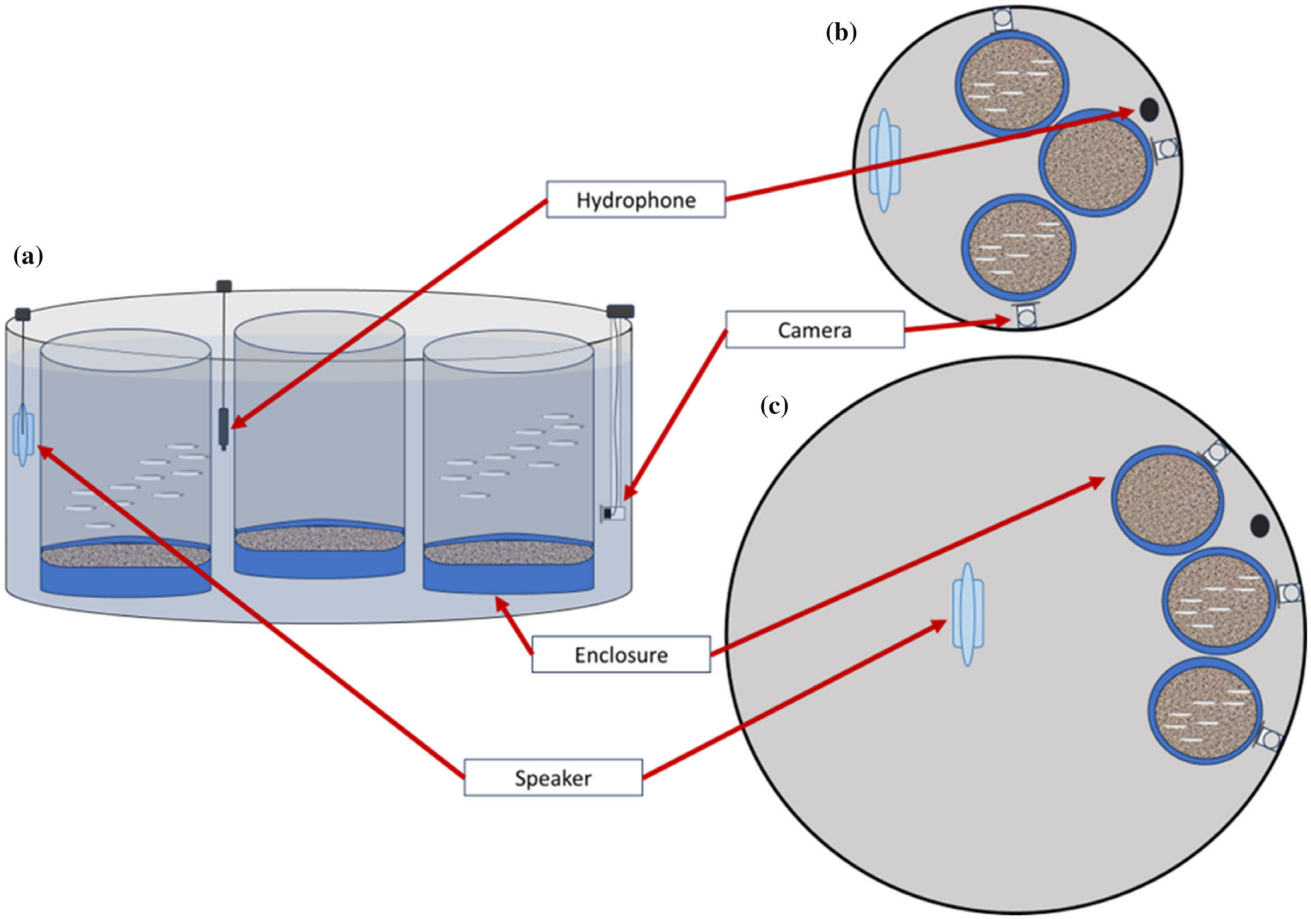
Pacific sand lance experimental tank schematic showing schools in (a) elevation, and (b) and (c) plan view depicting placement of semi‐permeable enclosures (1 m diameter). Approximate tank sizes are shown in (b) indoor, 2 m diameter, and (c) outdoor, 4 m diameter.

Due to logistical reasons and tank availability, there were two large concrete outdoor tanks (~11,300 L, 0.9 m height × 4 m diameter) shaded by a gazebo, and two small indoor concrete tanks (~2827 L, 0.9 m height × 2 m diameter) with floor to ceiling windows on one side so all tanks experienced the same photoperiod. Additionally, we had to run experiments on all schools simultaneously as we had a limited time window in which to use the tanks. We filled the bottom of each enclosure with ~10–15 cm of coarse sand from Jackson Beach (0.5 mm grain size) to allow PSL to bury. The flow‐through water system continuously supplied unfiltered seawater to each tank from the mouth of Friday Harbor from approximately 5 m deep which mirrored natural conditions (~10°C) and kept fresh cold seawater constantly moving though to maintain water quality and temperature and provided zooplankton which we supplemented on alternate days with live‐caught zooplankton collected locally using a plankton net (1 m diameter, 200‐μm mesh). All large tanks held 3 experimental enclosures, though only 2 ever housed fish, due to the fact that we were only able to catch enough fish to fill 8 enclosures, rather than the planned 12 (Figure 1). Each enclosure contained two cameras (ELP 2 megapixel IR webcam, Ailipu Technology 142 Co., Ltd., Guangdong, China): one underwater facing laterally, and one above the tank facing down. Each tank contained one underwater speaker (Lubell UW30, Lubell Labs Inc., Ohio, USA) which played a noise treatment (different playback for each tank) as well as an associated computer (Dell OptiPlex 7050 Core i5, running Windows 10 Pro OS), which started and stopped all noise playbacks and video recordings using Task Scheduler. We played all playbacks on Media Player (part of the Windows 10 OS) and recorded video using OBS studio (v. 30.0.2; Bailey and OBS Project Contributors, 2017). Although Lubell UW30 speakers only have a frequency range of 80–1300 Hz which excludes the very low frequencies usually produced by anthropogenic noise that fish can still detect (e.g., as low as 30 Hz for many fish species; Slabbekoorn et al., 2010), these speakers are standard in playback experiments with fish (e.g., Rojas et al., 2023; Xu et al., 2025), and the only ones we had access to for laboratory experiments. Additionally, PSL appears to be most sensitive to sounds between 50 and 350 Hz (Strobel & Mooney, 2012), mostly (~73%) within the range of the UW30 speaker. In this case, we would expect that any effect of noise we see on fish might be less than what we see in the wild as some frequencies are under‐represented. Therefore, although not replicating the sounds fish would experience in the wild, we still believe that this experiment provides an important first step to understanding how anthropogenic noise impacts fish behaviour.

As fish were wild‐caught, like in other wild‐caught sound studies, PSL were almost certainly exposed to some amount of noise, specifically boat noise, during their lifetime. However, as our experiment only exposed them to noise for 10 days, and as they had a week long period of no noise exposure before playbacks began, we think that any effects we see during the study are primarily a result of our playbacks rather than previous exposure or cumulative exposure.

Similarly, although each school varied in size and composition, based on our measured subsample, the variation in school composition was likely very small, with schools being made up of roughly the same size and age fish, and unlikely to greatly impact burying and startling behaviour. Additionally, while prey abundance likely differed across tanks, as we could not measure the amount of copepods we released into the tanks, nor the amount freely entering the tanks through the flow‐through system, we specifically did not record behaviour around feeding times to avoid changes in behaviour due to feeding. Outside of feeding times, fish all had access to the same water system, and food abundance. Finally, although the indoor and outdoor tanks were exposed to sunlight a bit differently, especially the wind farm tank, all tanks had access to a similar amount of sunlight (i.e., all tanks were under a roof), fish in control tanks did not appear to be affected by the overall amount of light (see results) and the observation times were at least 3.2 h after civil twilight ended and 2.5 h before civil twilight began (i.e., during full daylight) when fish should be exhibiting normal daytime behaviour. Therefore, we think that the difference in light was not enough to alter fish behaviour during the observational periods.

### 2.3 Acoustic treatments and experimental trials

After placing PSL in their enclosures in one of the four experimental tanks, fish were acclimated for 7 days with no playbacks or any disturbance. After the acclimation period, we simultaneously commenced playbacks in each tank for 11 consecutive days. Each tank received only one of the following acoustic treatments: (1) boat noise (2) wind farm noise, (3) pile driving noise or (4) silence (the control). Playbacks had a maximum dB re 1 μPa that did not exceed 140 dB re 1 μPa, (loud enough to be discernible above background noise levels but not enough to cause physical damage) (Popper & Hawkins, 2012; van der Knaap, Ashe, et al., 2022). Minimum (silence /ambient) conditions were 99 dB re 1 μPa (calculated: frequency range 0–24 kHz, over 3 s), and maximum conditions were 132 dB re 1 μPa (wind farm, calculated: frequency range 0–24 kHz, over 16 s), 138 dB re 1 μPa (pile driving, calculated: frequency range 0–24 kHz, over 27 s) and 138 dB re 1 μPa (boat noise, passing calculated: frequency range 0–24 kHz, over 15 s), (background, calculated: frequency range 0–24 kHz, over 11 s; received levels verified by calibrated SoundTrap STD300 hydrophones; Ocean Instruments NZ, Auckland, New Zealand; calculations using PAMGuide; Merchant et al., 2015). We determined the loudest area fish experienced by testing five locations in each enclosure (the centre, the closest and farthest from the speaker, and two at each edge perpendicular to the closest and farthest from the speaker—in four ‘corners’ of the circular enclosure), and dB levels varied across tanks, but within tanks, fish experienced similar levels, as sound tended to be loudest at the edge closest to the speaker and quietest at the edge farthest from the speaker (Figure S1). There was no difference in the control tank, the largest difference in the loudest and quietest area occurred in the wind farm condition (~30 dB re 1 μPa) and smaller differences in boat noise and pile driving tanks (boat noise passing ~20 dB re 1 μPa, boat noise background ~10 dB re 1 μPa, pile driving ~5 dB re 1 μPa). We checked sound bleed between tanks using the hydrophone and detected none. We created recordings using Audacity open‐source sound editing software (v3.2.2; Muse Group & Contributors). Playbacks were altered from natural conditions due to the concrete tanks, and most playbacks had peak amplitudes higher than source sounds (i.e., just above 100 Hz) and often thinner peaks (Figures S3–S5). Due to speaker noise in all tanks, there was a peak of energy at 2 kHz (Figures S2–S5). The UW30 speaker has a uniform frequency response between 80 and 1300 Hz, which resulted in slightly lower dB levels at lower frequency compared to recordings (Figures S3–S5). Control audio (i.e., Audacity‐generated silence) was played in the tank whenever anthropogenic noise playback (i.e., boat noise, pile driving and wind farm noise) was off. Due to restricted availability of noise recordings, we chose the recordings that were the best example of the sound for each treatment. Although we did not combine sounds from many different sources, the sounds we created were indicative of that type of sound source and likely representative of that type of sound in the wild. Additionally, as we had all fish from each treatment in one tank, we could not play back different audio files from different sources, so the sounds are pseudoreplicated. Speakers were placed outside the enclosures on the far side of the tank from the enclosures (small tanks) or in the centre of the tank (large tanks) facing the enclosures (Figure 1).

#### 2.3.1 Control

Silence generated by Audacity (Figure S2; Sound Exposure Level (SEL) = 98.18 dB re 1 μPa2s).

#### 2.3.2 Wind farm noise

Pervasive and chronic noise, occurring on average 12 h per day, 6 days per week (Table 1), with a dominant frequency response ranging from ~20 to 200 Hz, but no higher than 1 kHz (Tougaard et al., 2020; SEL = 129.01 dB re 1 μPa2s; Figure S3). Recordings were taken with a DSG loggerhead at the Utgrunden wind farm in the Baltic Sea (Sigray & Andersson, 2011) from 0.5 m above the sea floor, and we produced playback files from a 13 s file, which was repeated to create a 2‐h playback file.

**TABLE 1:** The (a) days and (b) hours that noise playback was on (days are shown as days in May, hours are shown from 03:00 to 21:00, all times outside shown times were silent).

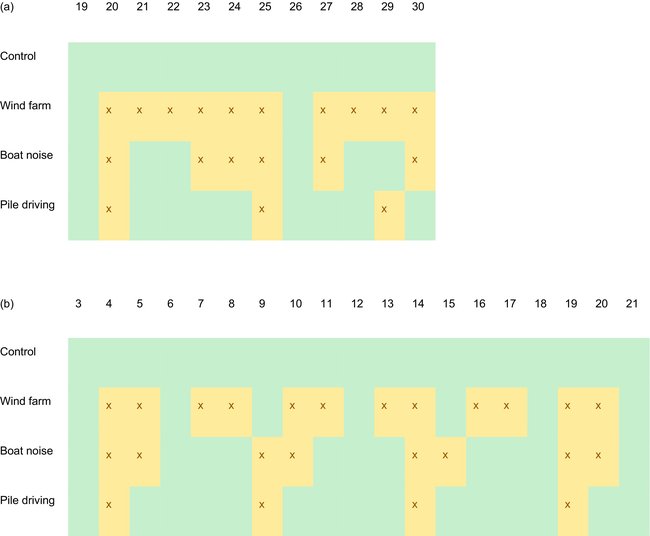

*Note*: Noise playback is indicated by an ‘x' and silence playback is indicated by an empty space. For our animal care protocol, noise was never on for more than 2 h at a time without at least a 1‐h break in between, and no noise was played back overnight (a minimum of 8 h).

#### 2.3.3 Pile driving noise

Intense, episodic noise consisting of ~1 strike/s for 60 min, occurring on average 4 times per day, 3 days per week (Table 1), with frequency range of 10 Hz – 500 Hz (dominant response 20–200 Hz; Bailey et al., 2010; SEL = 131.07 dB re 1 μPa2s; Figure S4); recorded ~300–500 m from the closest pile from a depth of 19.5 m–25.5 m, during monopile construction in the Dutch North Sea (provided from the APELAFICO project; Hubert et al., 2024). Fifteen‐minute consistent full‐strength pile driving noise (four repeats) created a 1‐h file, faded in (0–100% power) over the first 45 min to simulate typical ramp‐up.

#### 2.3.4 Boat noise

Variable noise occurring on average 6 h per day, 4 days per week and consisting of slow ramping up and down, of variable duration, with frequency range ≤ 10 Hz −30 kHz (Pine et al., 2018; SEL = 133.5‐ dB re 1 μPa2s when boats were passing; SEL = 119.29 dB re 1 μPa2s during ambient noise; Figure S5). Sound files included 2 h of ambient noise recorded from hydrophones situated in and around Vancouver Harbour (SMRU, 2019), combined with individual boat passes (10 different boat passes; ~1 pass every 10 min). Sounds came from hydrophones sitting 1.5 m from the ocean floor, in Burrard west (loudest, 28–41 m deep), English bay (Intermediate; 55–62 m deep) and Indian Arm (quietest, 69–71 m deep) and were combined to create the boat noise audio file. Boat passes included small (e.g., speedboats; higher frequency sound) and large (e.g., large ferries; lower frequency sounds; Figure S4) vessels.

### 2.4 Particle motion

Although particle motion (also called sound velocity level—SVL) is an important way that fish, especially those without swim bladder such as PSL, sense sound in their environment (Hazelwood et al., 2018; Rogers et al., 2021; Strobel & Mooney, 2012), due to a lack of access to particle motion detectors, we could not record particle motion in the water (via neutrally buoyant particle motion detector) or sand (substrate vibration via a vibrometer) during our experiment. However, particle motion tends to be comparable to sound pressure level (SPL) when travelling in water (Campbell et al., 2019; Popper & Hawkins, 2018; Sigray & Andersson, 2011), though this can differ inside tanks where SPL and SVL often decouple due to oscillating amplitudes and resonance; tank shape and material impacts how and the degree to which this happens (Gray et al., 2016; Hubert et al., 2016). These differences have been suggested to result in both higher (i.e., have excess SVL; Hubert et al., 2016; Parvulescu, 1967) or lower SVL values than expected when compared to SPL depending on the setup (Campbell et al., 2019; Hubert et al., 2016).

### 2.5 Predator presentations & feeding

In addition to playbacks, we conducted simulated predator encounters by rapidly submerging (~2 s) a black plastic model of a Marbled Murrelet (*Brachyramphus marmoratus*) in a diving position into the surface of the water in each enclosure. Predator presentations occurred 3–4 times in each enclosure throughout the experimental trial period. Behavioural responses to these simulated predator encounters are not included in this experiment due to small sample size and a different focus. The anti‐predator behavioural responses observed and recorded in this study were taken at standardized times throughout the day, specifically avoiding the times of day around the predator encounter simulations to reduce any potential impact on PSL behaviour. Predator presentation days were included as a fixed effect in the models (predator event) to detect any impact. Similarly, fish received food once every 2 days. As the food was primarily acquired via plankton net towing on that day, we could not control the exact amount of food each tank received and so did not include feeding behaviour in the analysis. However, as feeding might impact other behaviours, we avoided taking behavioural measures close to feeding times to reduce any potential impact on PSL behaviour, and feeding days were included as a fixed effect in the models (feeding) to detect any impact.

### 2.6 Data collection

We used Solomon Coder (v. beta 19.08.02) behavioural coding software, to record fish behaviour and created an ethogram. We then chose two anti‐predator behaviours that occurred often enough to determine how playbacks of anthropogenic noise affect anti‐predator behaviour: burying and startling (Table 1). To determine burying behaviour, we recorded the relative number of fish in the water column (i.e., not buried; number of swimming fish/ total number of fish in enclosure—we could not triangulate fish position in the water column accurately since there was only one submerged camera in each tank, and so only included buried and non‐buried rather than height in the water column). To determine startling behaviour, we recorded the proportion of startled fish in the water column (number of startled fish/number of fish in water column). Counts of relative number of fish in the water column and startling behaviour were documented in 1‐min intervals for 2 min in each treatment (Table 2). Due to limited visibility during periods of low to no light, we could not code responses either in the early morning or late night. We limited our observation times to between 9:00 and 19:15; this was a minimum of 3.25 h after the start of civil twilight (morning) and 2.50 h before the beginning of civil twilight (evening), increasing throughout the 10 days. Of the 576 1‐min recordings, we could not process 23, resulting in 553 observations across 4 tanks and 8 enclosures (control: enclosure 1 = 72, enclosure 2 = 72; boat noise: enclosure 1 = 72, enclosure 2 = 72; pile driving: enclosure 1 = 64, enclosure 2 = 64; wind farm: enclosure 1 = 70, enclosure 2 = 67).

**TABLE 2:** Times when Pacific sand lance anti‐predator behaviour was coded by a trained observer in each acoustic environment from 19 to 30 May 2023.

Times coded^a^	Time	Acoustic environment
09:01	Morning	Boat noise, pile driving
09:02		
10:06		Wind farm, silence
10:07		
14:05	Afternoon	Boat noise, pile driving, wind farm, silence
14:09		
19:05	Evening	Boat noise, pile driving, wind farm, silence
19:09		

^a^ Represents times when playbacks were on for the respective treatment, to provide comparison of PSL behaviour between days when noise playbacks were on or off.

### 2.7 Statistical analyses

We used the lme4 package (Bates et al., 2015) in R (v.4.3.2, R Core Team, 2023) for all statistical analyses. We used a binomial generalized linear mixed effects model to quantify whether the relative total number of PSL in the water column (response variable) differed in response to noise condition. Explanatory variables included whether the playback was on or off (playback state (discrete): on, off, control), time of day (time (discrete): morning, afternoon, evening), number of consecutive days exposed to noise or silence (exposure (continuous): 0–10, with day 0 being the first date of recorded behaviour and silent playback in all tanks), days when predator presentations occurred (predator event (discrete): yes, no), days when fish were supplemented with food (feeding (discrete): yes, no), and the cloud cover to account for differences in light conditions that could impact behaviour. We obtained cloud cover data (proportion cover, hourly temporal resolution) from the ERA5 time series from Copernicus Climate Change Service (2025) and used the 0.25 × 0.25 degree spatial grid point closest to Friday Harbor Laboratories (in Griffon Bay, San Juan Island, lat: 48.50, lon: −123.00, ~5 km south of the location of the tanks, lat: 48.55 long: −123.01). We included enclosure ID and trial day as random intercepts in the model to account for repeated measures within each school and temporal autocorrelation, respectively (Figures S6–S9; Tables S1, S3, S5, and S7), though we could not account for the pseudoreplication caused by both schools being in the same larger tank. We included both feeding days, and predator presentations in our models, though we did not code data during those times due to small sample size (predator presentations) and the potential variability of food quantities (feeding); both events had the potential to impact behaviour on the days those events occurred. For boat noise, we included the interaction between time and playback state as well as time and exposure since boat noise had multiple days on and off, whereas pile driving was only on for 1 day at a time and wind farm only had one noise‐off day so we only included the playback state and time of day interaction for these two conditions. We ran a type II Wald Chi‐Square test on the model to test the significance of each explanatory variable in the model (Tables S2, S4, S6 and S8).

We used a binomial generalized linear mixed effects model to investigate whether the proportion of startled PSL (response variable) differed in response to noise condition, with playback state, time, exposure, predator event and feeding as explanatory variables. Enclosure and trial day were included as random intercepts in the model. Interactions were as stated for the previous model (Tables S9, S11, S13 and S15; Figures S10–S13). We ran a type II Wald Chi‐Square test on the model to test the significance of each explanatory variable in the model (Tables S10, S12, S14 and S16). Diagnostic plots (i.e., QQ plots; Tremblay et al., 2020) showed that models were not well fitted with slightly skewed residuals and quantiles that deviated from the line towards each end (Figures S10–S13); however, due to the structure of the data, we have been unable to find a better statistical approach. Nonetheless, these startle response models provide valuable information and generally accurate trends on startling behaviour in PSL, though under‐predicting low values and over‐predicting high values may result in exaggerated estimates.

We also ran two models, one for proportion of fish in the water column and one for startle response for only control (no playback response) to determine if there were effects of any of the included factors (time of day, exposure, predator, feeding and cloud cover) on the behaviour of fish in non‐noisy conditions.

### 2.8 Ethical note

All fish were caught and transported under the University of Washington's Friday Harbor Collections Licence, and experiments were reviewed and approved under the University of Washington's Institutional Animal Care and Use Committee (IACUC) protocol 4238‐19 and the University of Victoria's Animal Care Committee's approved Animal Use Protocol (AUP) 2022‐022. This was supported through an Inter‐Institutional Memorandum of Understanding for Animal Care and Use Oversight. This research included exposing animals to noisy environments in the lab. Noise can cause increases in stress in animals, and injury or death if too loud. In our experiments, our animals were only subjected to noise for a maximum of 11 days, and never had noise playing for more than 2 h at a time without at least a 1‐h break in between, and no noise was played back overnight (a minimum of 8 h). All noise was played back at <140 dB re 1 μPa, loud enough to hear over the background tank noise and at similar levels as they may encounter in areas like Vancouver Harbour (SMRU, 2019) but not loud enough to result in permanent damage to hearing or injury (Popper & Hawkins, 2012; van der Knaap, Ashe, et al., 2022). Additionally, this paper discusses one portion of a larger experiment that included simulated predator presentations which lasted a few seconds, after which fish returned to normal behaviour within 5–10 min. These presentations were only done four times over the course of the 11 experimental days. As part of the larger experiment's conclusion, a portion of fish were caught and humanely euthanized using MS‐222 to take lethal measurements (i.e., energy density, see Carlson et al., 2025) while the rest were released back at the location they were caught. This research adheres to the ASAB/ABS Guidelines for the ethical treatment of nonhuman animals in behavioural research and teaching.

## 3 RESULTS

### 3.1 Ethogram

After spending ~45 h watching PSL videos, we generated a PSL ethogram that includes 11 behaviours. We recorded five known behaviours including: head exposure, swimming, burying, startling and feeding (i.e., *A. americanus*, *A. japonicus*, *A. heian* and *A. personatus*; Amiya et al., 2023; Haynes et al., 2007; Meyer et al., 1979; Winslade, 1974), and identified six behaviours not previously described: twitching, continuous feeding, biting, contorting, gaping and freezing (Table 3). Due to the rarity of many of the behaviours, we could not include them in statistical analysis when examining PSL response to noise.

**TABLE 3:** Ethogram of captive Pacific sand lance (*Ammodytes personatus*) during 11 days of experimental trials.

Behaviour	Description
Swimming	Lateral undulations that increase in amplitude caudally (anguilliform locomotion; Gidmark et al., 2011)
Startle/escape response	Sudden burst of swimming speed at an altered angle relative to pre‐startle direction
Burying	Rapidly burrowing head first into the sand
Emerging	Returning into the water column head first from the sand
Twitching	Rapid movement/jerk of the anterior portion of body perpendicular (either towards left or right side) to the posterior portion of the body
Jaw movement	Rapidly and consecutively opening and closing mouth while swimming
Biting	One fish attempts to or successfully bites another fish
Contorting	Slow dorsal‐ventral bending of the head and tail in the same direction
Gaping	Mouth remaining open while swimming
Head exposure	Head of fish exposed to water column while remainder of fish is buried
Freezing	Lying unmoving on the surface of the substrate, with one side of body in contact with the substrate

### 3.2 Relative number of fish in water column

In the control condition, time of day impacted how many fish were in the water column with fewer fish present as the day progressed (χ^2^ = 79.43, *p* < 0.001; Figure 2), no other factors impacted burying behaviour (exposure: χ^2^ = 0.41, *p* = 0.522; predator: χ^2^ = 1.03, *p* = 0.311; feeding: χ^2^ = 0.20, *p* = 0.652; cloud cover: χ^2^ = 0.98, *p* = 0.322; Random effects: day: variance = 0.03, std. dev. = 0.16, enclosure: variance = 0.11, std. dev. = 0.34; Tables S1 and S2).

**FIGURE 2:**
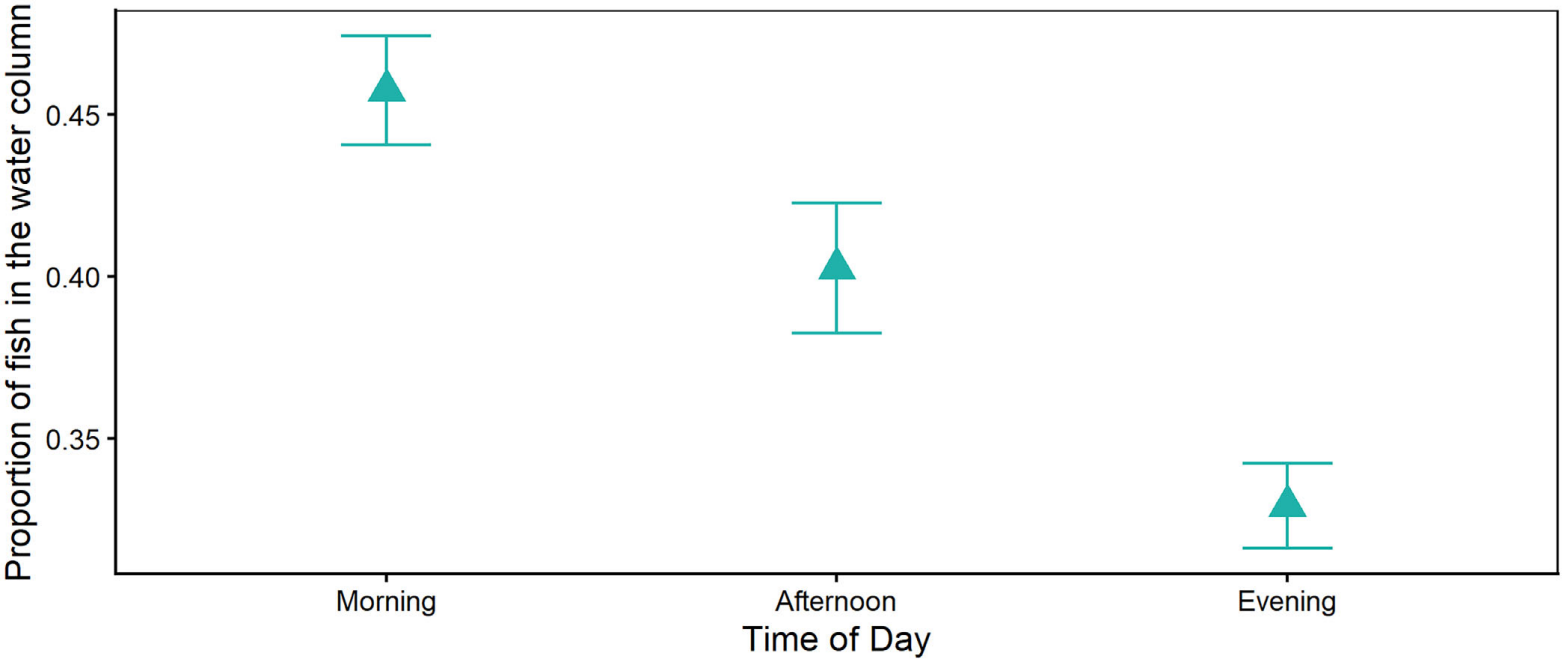
Relative number of Pacific sand lance (*Ammodytes personatus*) in the water column (mean ± std. error) in the control acoustic environment across the day (i.e., morning, afternoon and evening) over 12 days.

In the boat noise acoustic environment, there was a significant interaction between playback state and exposure (χ^2^ = 34.21, *p* < 0.001), and between playback state and time (χ^2^ = 44.83, *p* < 0.001), and the effect of cloud cover (χ^2^ = 7.33, *p* = 0.007) on the relative number of fish in the water column (Figure 3; Tables S3 and S4). Predator event (χ^2^ = 0.80, *p* = 0.371) and feeding (χ^2^ = 0.04, *p* = 0.846) had no significant effect (random effects: day: variance = 0.03, std. dev. = 0.17, enclosure: variance = 0.05, std. dev. = 0.23).

**FIGURE 3:**
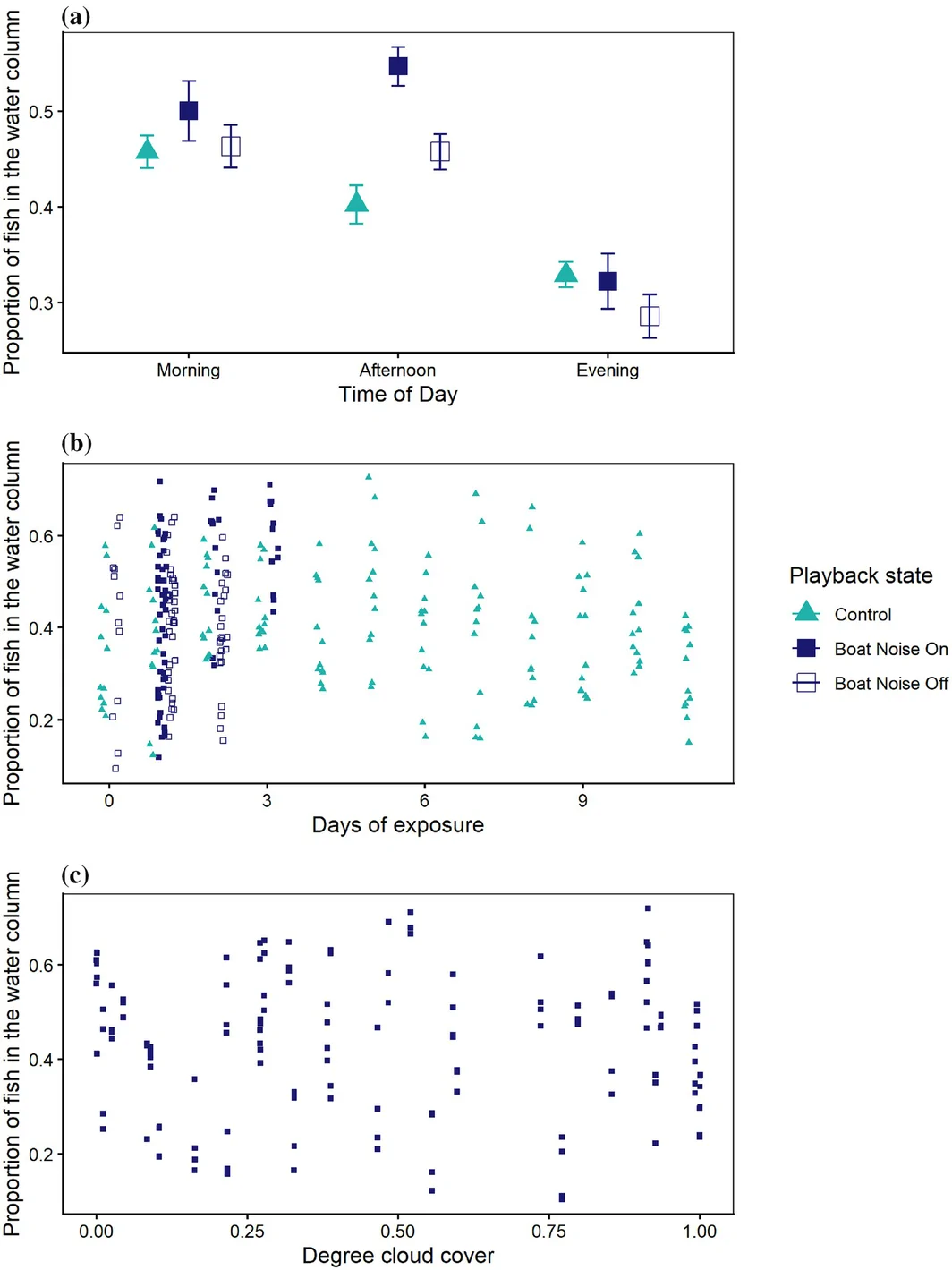
Relative number of Pacific sand lance (*Ammodytes personatus*) in the water column (mean ± std. error) in the control and boat noise acoustic environment showing (a) the interaction between playback state (i.e., control, on, and off) and time (i.e., morning, afternoon and evening), and (b) the interaction between playback state and exposure (i.e., number of consecutive days of noise or silence in the trial period) and (c) across varying degrees of cloud cover (boat noise data combined). Fish were exposed to four 2‐h boat noise playbacks for eight out of 12 experimental days (Table 1).

In the pile driving acoustic environment, the interaction between playback state and time of day had a significant effect on the relative number of fish in the water column (χ^2^ = 16.13, *p* = 0.003), as did exposure (χ^2^ = 7.69, *p* = 0.006); while predator event (χ^2^ = 2.54, *p* = 0.111), feeding (χ^2^ = 0.23, *p* = 0.630) and cloud cover (χ^2^ = 0.08, *p* = 0.772) did not (Figure 4; random effects: day: variance = 0.05, std. dev. = 0.23, enclosure: variance = 0.06, std. dev. = 0.24; Tables S5 and S6).

**FIGURE 4:**
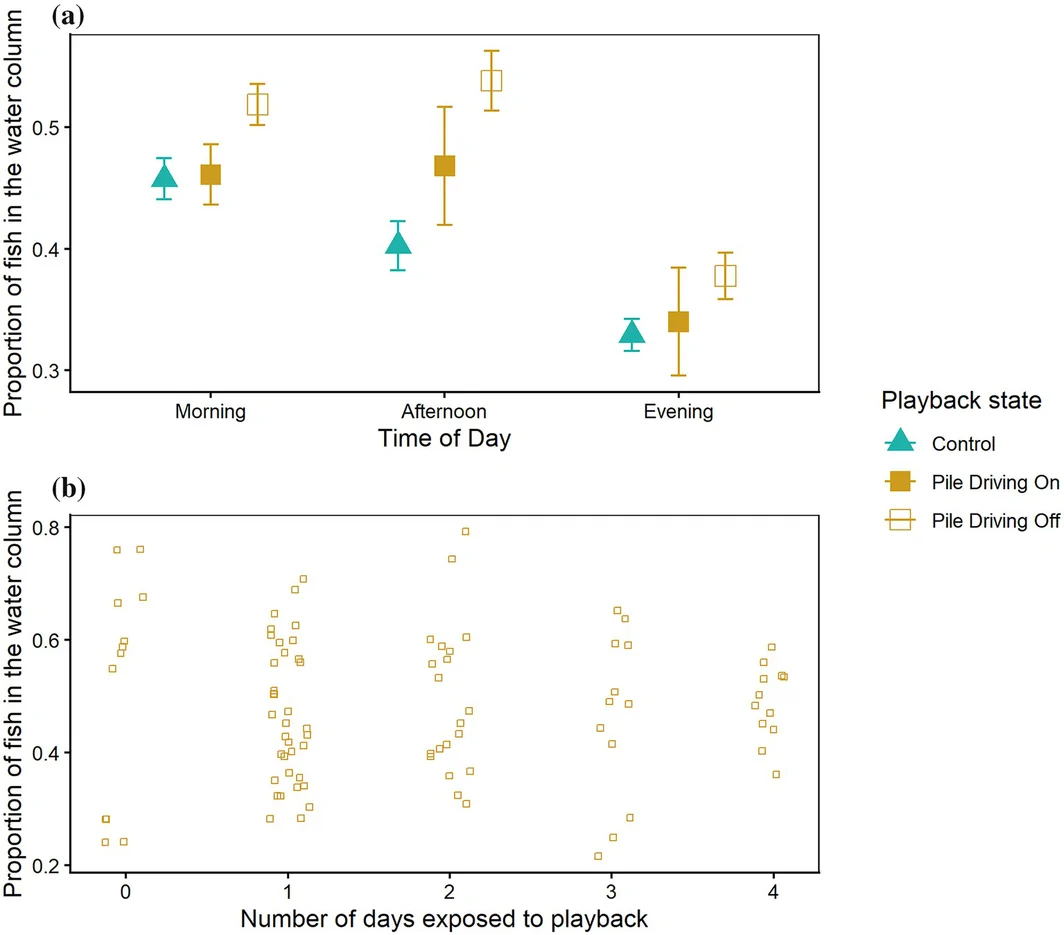
Relative number of Pacific sand lance (*Ammodytes personatus*) in the water column (mean ± std. error) showing (a) the interaction between playback state (i.e., control, on and off) and time (i.e., morning, afternoon and evening) and (b) across exposure period (only pile driving days off). Pile driving noise was played for 1 h at a time, four times a day when on, and played every 5 days for 12 days (Table 1).

In the wind farm acoustic environment, the interaction of time and playback state (χ^2^ = 288.04, *p* < 0.001), exposure (χ^2^ = 172.25, *p* < 0.001) and feeding (χ^2^ = 68.21, *p* < 0.001) had a significant effect on the relative number of fish in the water column, while predator (χ^2^ = 0.89, *p* = 0.346) and cloud cover (χ^2^ = 2.56, *p* = 0.110) did not (Figure 5; Random effects: day: variance = 0.56, std. dev. = 0.75, enclosure: variance = 0.11, std. dev. = 0.33; Tables S7 and S8).

**FIGURE 5:**
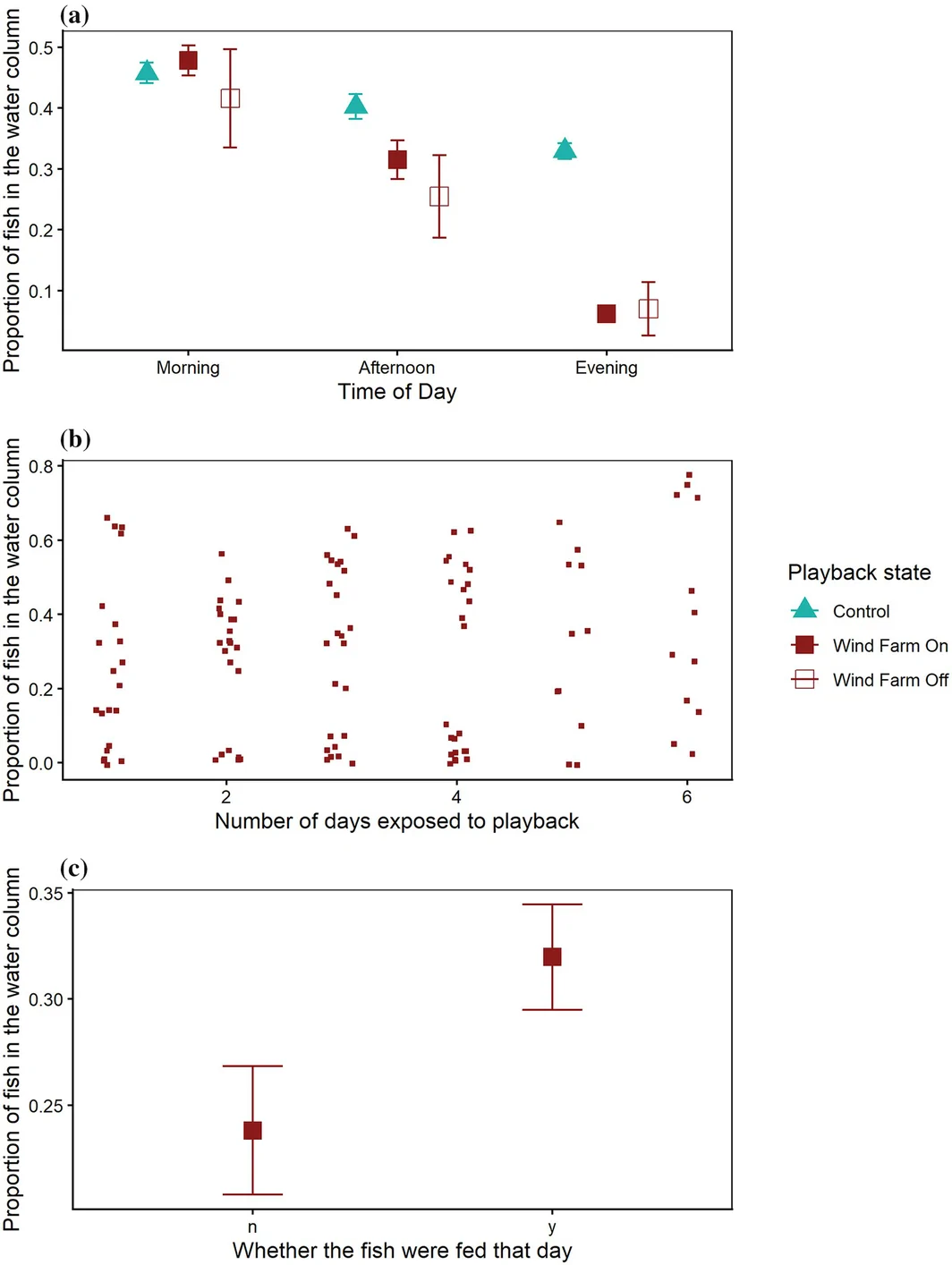
Relative number of Pacific sand lance (*Ammodytes personatus*) in the water column (mean ± std. error) in the control and wind farm acoustic environment at (a) the interaction between playback state (i.e., control, on and off) and time (i.e., morning, afternoon and evening), (b) number of consecutive days exposed to noise or silence (wind farm on days) and (c) days fed (wind farm data combined). Fish were exposed to six 2‐h wind farm playbacks for 10 out of 12 experimental days.

### 3.3 Proportion of startled fish

In control conditions, whether or not fish experienced a predator simulation on that day affected startling behaviour (χ^2^ = 4.79, *p* = 0.029; Figure 6), no other factors did (time of day: χ^2^ = 0.72, *p* = 0.697; exposure: χ^2^ = 0.95, *p* = 0.330; feeding: χ^2^ = 0.96, *p* = 0.326; cloud cover: χ^2^ = 0.42, *p* = 0.519; Random effects: day: variance = 0.28, std. dev. = 0.53, enclosure: variance = 0.02, std. dev. = 0.15; Tables S9 and S10).

**FIGURE 6:**
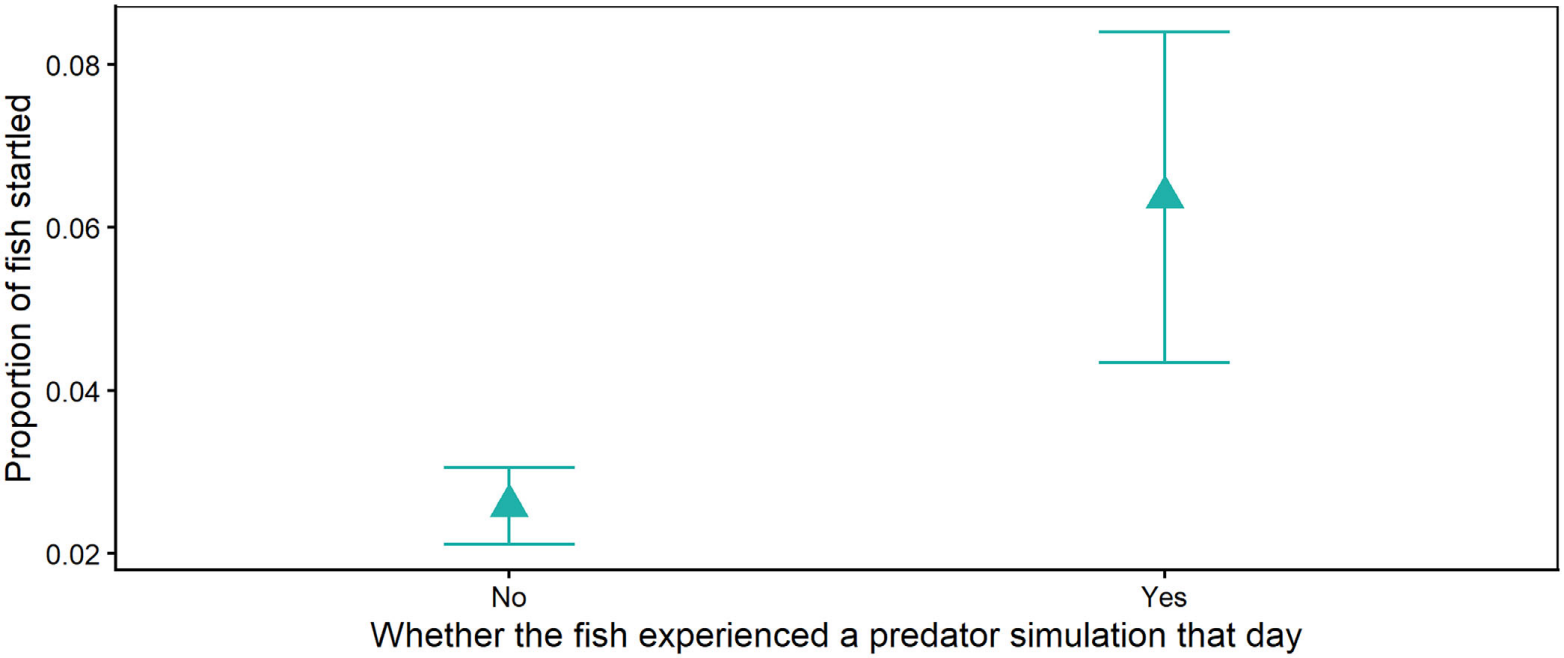
Proportion of startled Pacific sand lance (*Ammodytes personatus*; mean ± std. error) in the control treatment on days that they were or were not exposed to a simulated predator encounter. Experiments occurred over 12 days.

In the boat noise acoustic environment, there was a significant interaction between playback state and exposure (χ^2^ = 15.86, *p* < 0.001), between playback state and time (χ^2^ = 29.77, *p* < 0.001) and across different degrees of cloud cover (χ^2^ = 8.55, *p* = 0.003) for the proportion of startled fish, while predator event (χ^2^ = 3.38, *p* = 0.066) and feeding (χ^2^ = 0.34, *p* = 0.561) had no significant effect (Figure 7; Random effects: day: variance = 0.68, std. dev. = 0.82, enclosure: variance = 0.22, std. dev. = 0.47; Tables S11 and S12).

**FIGURE 7:**
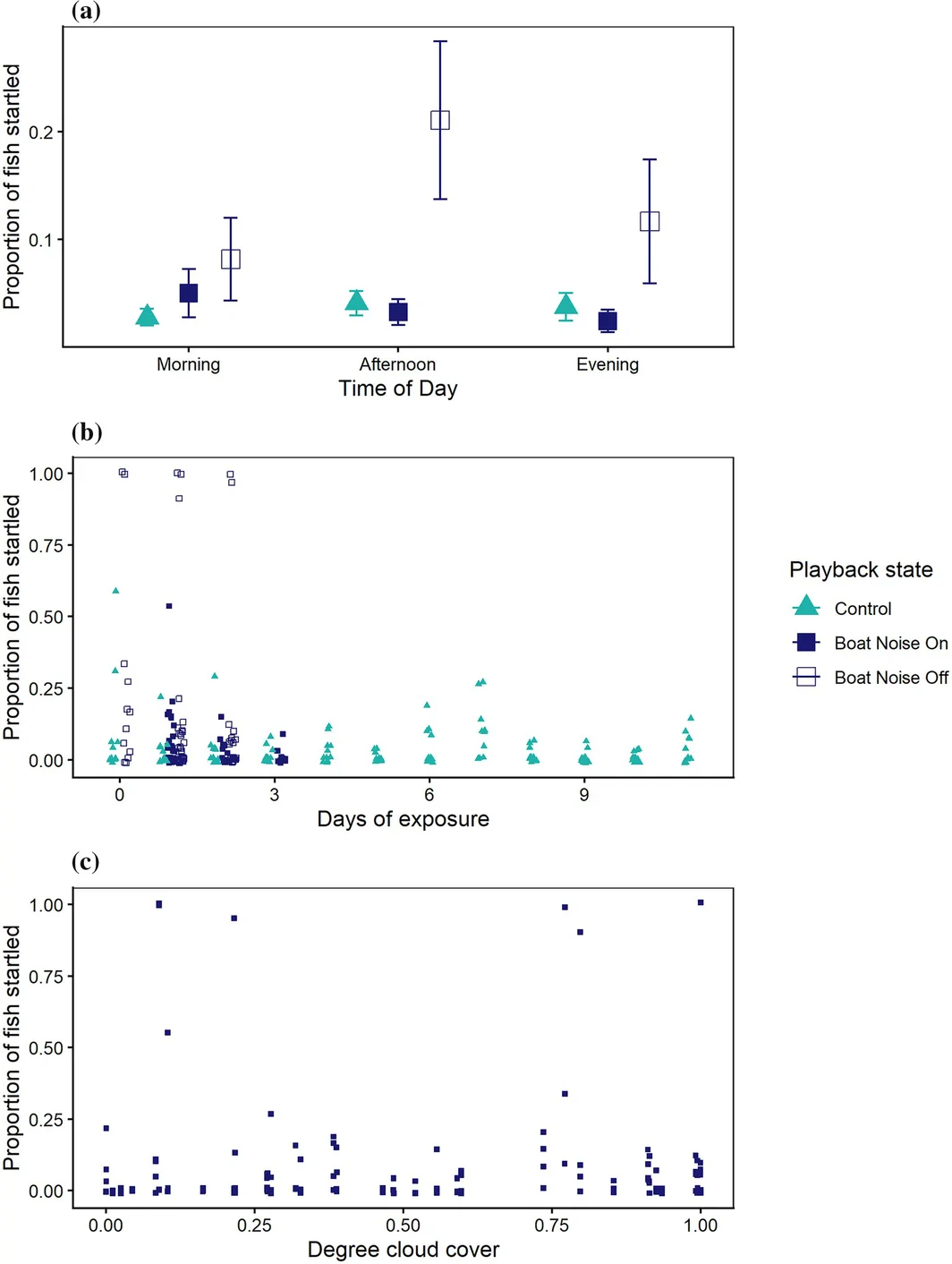
Proportion of startled Pacific sand lance (*Ammodytes personatus*; mean ± std. error) in the control and boat noise acoustic environment showing (a) the interaction between playback state (i.e., control, on and off) and time (i.e., morning, afternoon and evening), (b) on consecutive days of noise or silence in the trial period and (c) across different degrees of cloud cover (boat noise data combined). Fish were exposed to four 2‐h wind farm playbacks for 6 out of 12 experimental days.

In the pile driving acoustic environment, the interaction between playback state and time (χ^2^ = 18.96, *p* = 0.001) and cloud cover (χ^2^ = 10.20, *p* = 0.001) had a significant effect on the proportion of startled fish, while exposure (χ^2^ = 0.04, *p* = 0.836), predator (χ^2^ = 2.04, *p* = 0.153) and feeding (χ^2^ = 0.01, *p* = 0.914) did not (Figure 8; Random effects: day: variance = 0.74, std. dev. = 0.86, enclosure: variance <0.01, std. dev. = 0.01; Tables S13 and S14).

**FIGURE 8:**
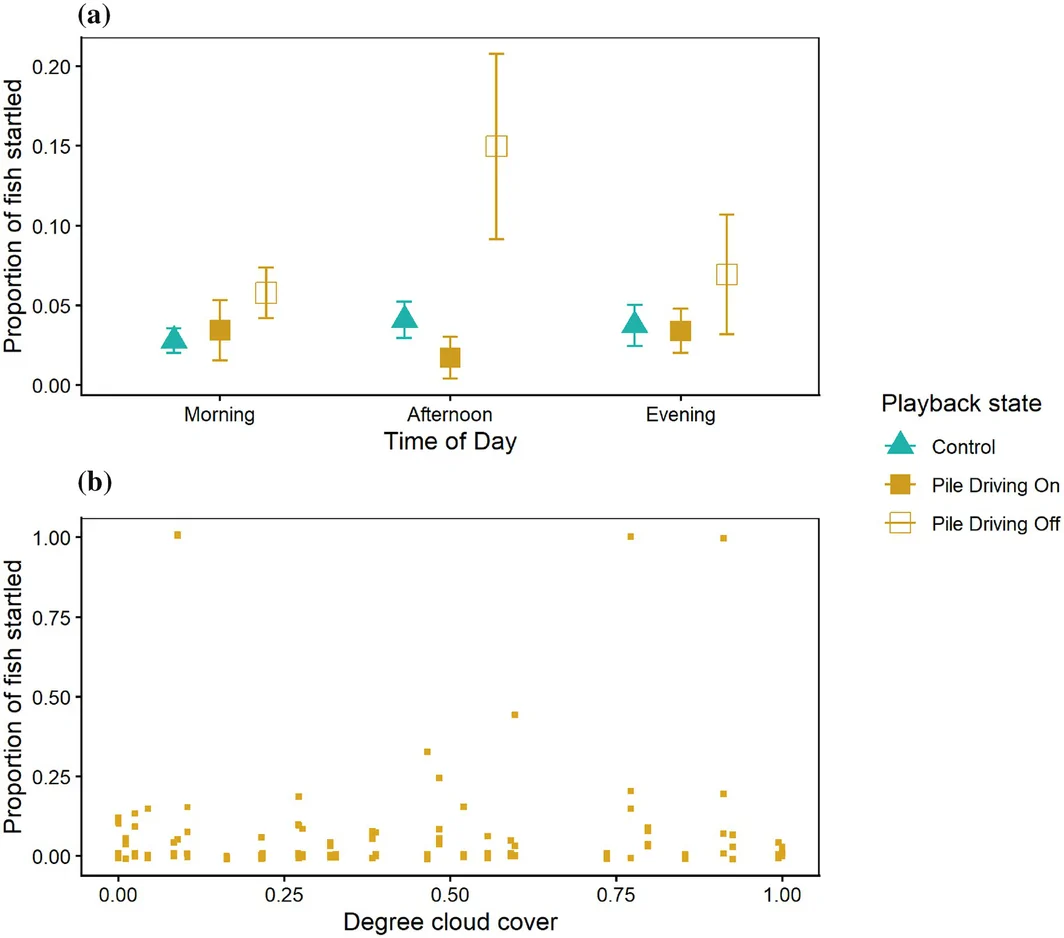
Proportion of startled Pacific sand lance (*Ammodytes personatus*; mean ± std. error) in the pile driving acoustic environment showing (a) the interaction between playback state (i.e., control, on and off) and time (i.e., morning, afternoon and evening) and (b) across different degrees of cloud cover (pile driving data combined). Fish were exposed to four 1‐h pile driving playbacks for 3 out of 12 experimental days.

In the wind farm acoustic environment, the interaction of playback state and time (χ^2^ = 31.52, *p* < 0.001), exposure (χ^2^ = 11.06, *p* = 0.001) and cloud cover (χ^2^ = 6.56, *p* = 0.010) had a significant effect on the proportion of startled fish (Figure 9). All other factors did not significantly affect the proportion of startled fish (predator: χ^2^ = 2.49, *p* = 0.115; feeding: χ^2^ = 0.18, *p* = 0.674; random effects: day: variance = 1.22, std. dev. = 1.10, enclosure: variance = 0.11, std. dev. = 0.33; Tables S15 and S16).

**FIGURE 9:**
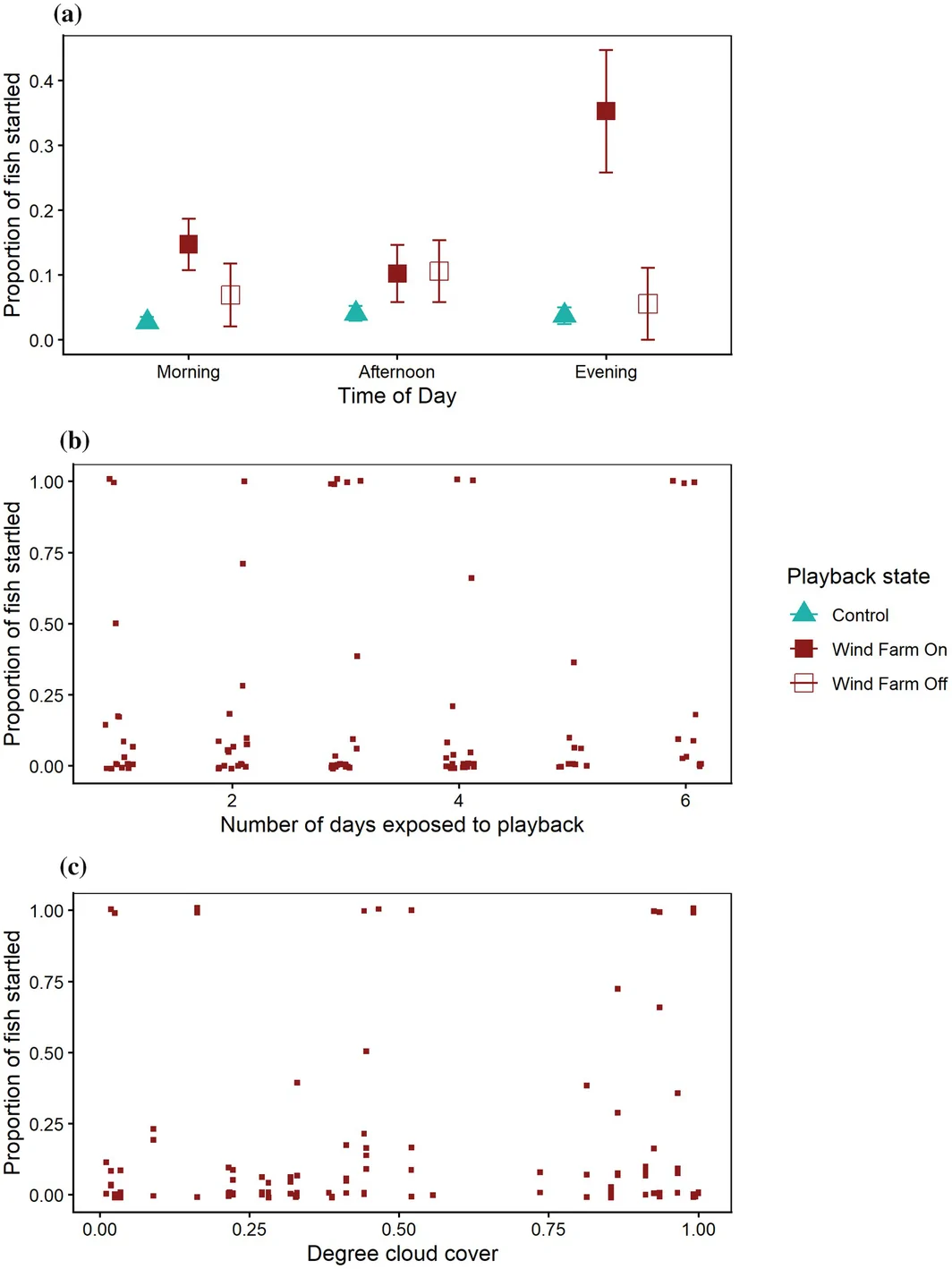
Proportion of startled Pacific sand lance (*Ammodytes personatus*; mean ± std. error) in the wind farm acoustic environment showing (a) the interaction between playback state (i.e., control, on and off) and time (i.e., morning, afternoon and evening), (b) number of consecutive days of exposure to noise or silence (wind farm on days) and (c) degree of cloud cover (wind farm data combined). Fish were exposed to six 2‐h wind farm playbacks for 10 out of 12 experimental days.

## 4 DISCUSSION

### 4.1 PSL ethogram

PSL have a wide range of behaviours they exhibit in captivity. To our knowledge, this study provides the first ethogram of captive PSL (Table 2). Despite impacts of captivity on behaviour (Jones et al., 2019; Simpson et al., 2015), laboratory studies provide controlled conditions in which behaviour can be recorded to an extent that might not be possible in an in situ experiment (Woods et al., 2022). We observed six behaviours not previously described including twitching, contorting, jaw movement, biting, gaping and freezing.

While uncommon, twitching occurred in all schools and is therefore likely not a result of exposure to noise. Twitching could be the result of a parasitic infection (Pers. Comm. Dr. Duguid and Dr. Carlson, 2024) that could be brought in from the wild with the fish or from unfiltered seawater or exacerbated through captive stress (Barber, 2007). Salmon exhibit behaviours to dislodge parasites such as jumping behaviour (Atkinson et al., 2018; Fagen, 2017) and twitching behaviour in PSL may serve a similar purpose. Twitching may also be similar to contorting, or slow dorsal‐ventral bending of the head and tail in the same direction, which is also seen in PSL and is similar to contorting behaviour in killifish, potentially a parasite‐related behaviour that makes them more susceptible to avian predators, thus perpetuating the life cycle of their trematode parasite (Lafferty & Morris, 1996). Many fish exhibit a range of aggressive behaviours across a range of contexts. Jaw movement, gaping and biting behaviours are likely signs of aggression as similar behaviours occur in zebrafish (*Danio rerio*; Kalueff et al., 2013). However, in the case of jaw movement and gaping, there were no obvious targets of aggression.

Freezing is, to our knowledge, only present in *Ammodytes* species. It is likely an acute stress response, as it was commonly observed after fish were collected in the field and after they were transferred to new tanks. Freezing was also seen in situ, both during fish release and when fish emerged from the sand on beaches to return to the water (Pers. Obs. NVC; Quinn & Schneider, 1991). On local beaches, in the wild, although there were many gulls, crows and shorebirds eating emerging fish, many PSL remained motionless after emerging, even though they were still alive (Pers Obs. NVC). Birds appeared to pursue PSL that were in the process of emerging, or had just emerged and were moving, not motionless, suggesting that freezing could be an anti‐predator response. In captivity, PSL exhibiting freezing behaviour remained motionless for minutes to hours at a time—even up to a day, which might provoke researchers to assume that the fish has died. Based on our experience with keeping PSL in captivity, we suggest waiting a minimum of 12–24 h before attempting to remove a motionless fish to reduce the potential of additional stress caused by capture.

### 4.2 Anti‐predator behaviour & response to playbacks of anthropogenic noise

Boat noise, wind farm noise and pile driving noise playbacks altered captive PSL behaviour. Yet, how noise affected PSL behaviour was not consistent across all acoustic environments suggesting that different sources of anthropogenic noise may affect fish differently. Although noise may similarly impact physiology (Carlson et al., 2025), our results suggest that the behavioural mechanisms through which this occurs could differ. Additionally, time of day had a consistent effect on PSL behaviour. In control conditions, PSL were most active in the water column in the morning, and activity decreased throughout the day. Fish in noise treatments tended to generally follow a similar trend, though to different degrees. In contrast, control fish had a relatively stable startle response throughout the day, while noise treatments generally increased the proportion of fish startling. These results also suggest that different noise sources may differentially impact the availability of PSL to predators (Carlson et al., 2026).

### 4.3 Proportion of fish in water column

There were relatively more PSL swimming in the water column in the afternoon during the pile driving (off) and boat noise (on) playback conditions compared to silence; (Figures 3a and 4a; Tables S3 and S5). Wind farm noise had the opposite effect, with similar numbers in the morning, but fewer fish in the water column in the afternoon and evening compared to the control (Figure 5a). In the pile driving and boat noise condition, the noise may have negatively impacted their foraging efficiency, potentially leading to increased foraging time to meet the same energetic requirement as in an environment when they were not exposed to noise (Ahrens et al., 2012; Purser & Radford, 2011). Noise may also distract, resulting in PSL ignoring or missing prey items (Chan et al., 2010). Playbacks of anthropogenic noise can also elicit stress and increase locomotor activity, reducing energy allocation to activities such as foraging, predator–prey interactions and reproduction as seen in spiny lobsters (*Palinurus elephas*; Filiciotto et al., 2014). Similar increases in swimming activity have been observed in fishes exposed to anthropogenic noise (Neo et al., 2018), which may reduce time allocated to refuge behaviour such as burying. Increasing time in the water column can be costly, as it increases individual predation risk, and can result in energetic deficits, making them lower quality prey (Carlson et al., 2025; Horkan & Baker, 2025; Simpson et al., 2016; Stahlman et al., 2011; Wale et al., 2013), even if they are temporarily more available.

In the case of wind farms, the chronic nature of the noise may have induced chronic stress in PSL, resulting in the depletion of energy reserves (Carlson et al., 2025; Santos et al., 2010; Wei et al., 2018). By remaining in the substrate, PSL can conserve energy (Ahrens et al., 2012; Baker et al., 2019; Horkan & Baker, 2025), which may be used in other stressful conditions aside from dormancy. This observation is supported by Carlson et al. (2025) who showed that exposure to wind farm noise resulted in PSL having lower energy density and reduced weight at length relative to boat noise, pile driving noise and silence. Aside from their noise effects, the physical presence of wind farms may also impact the availability of PSL prey, as wind farms have been shown to alter phytoplankton and zooplankton distributions (Daewel et al., 2022; Dorrell et al., 2022). Increased burying time can make PSL unavailable to many of their predators and may also result in lower quality fish. Consistent with this, there was a significantly higher proportion of PSL in the water column during feeding events than during non‐feeding events in the wind farm treatment (Figure 5c). As PSL in the wind farm treatment generally spent more time buried than those in the other treatments, these feeding events likely prompted fish to emerge from the substrate and enter the water column to exploit the increased food availability, thereby offsetting reduced foraging opportunities associated with time spent buried.

### 4.4 Startling behaviour

PSL startled more often in noise treatments than in controls, which stayed consistently low throughout. Startling in noise treatments was modulated by time of day, but differently across treatments. Boat and pile driving noise had similar temporal trends. They were similar to the control in the morning, increased to peak startling in the afternoon and decreased back towards the control in the evening. Both boat noise and pile driving noise had higher startle responses when noise was off compared to on. These responses were not consistent with our hypothesis, with the potential exception of wind farm noise. This result suggests that, rather than perceiving noise as a predator, exposure to pile driving noise and boat noise may have resulted in a twofold response. First, being in a noisy condition may increase overall stress resulting in heightened vigilance and anti‐predator responses (Cox et al., 2018), explaining increased startle responses in fish exposed to windfarm playbacks. However, while fish may quickly return to baseline behaviour after noise stops (e.g., opercular beat rate, Neledec et al., 2016, or swimming patterns, Neo et al., 2014), noise type can impact this recovery time (Neo et al., 2014). Additionally, there is little information about if recovery of vigilance and startle behaviour is similar, or takes longer to return to baseline. If it takes more than a few hours for fish to recover normal startling behaviour after being exposed to chronic noise then even when the noise stops, fish may retain a heightened startle response, partially explaining the response of boat noise and pile driving fish when noise is off. Additionally, when actively on, noise may also cause noise‐induced distraction (Chan et al., 2010), which has been observed in many fish species (Purser & Radford, 2011; Simpson et al., 2015; Spiga et al., 2017). Boat noise and pile driving noise could elicit a masking effect (i.e., obscuring acoustic cues of interest) in PSL, making it difficult to detect a potential predator (Herbert‐Read et al., 2017) resulting in a lowered anti‐predator response (compared to when noise is off). Noise‐induced stress can also impact startling responses (Anderson et al., 2011; Smith et al., 2004; Wright et al., 2007). Such stress can disrupt a fish's capacity to process sensory information, lowering the chance of detecting approaching predators, and therefore their startling response (Herbert‐Read et al., 2017; Kight & Swaddle, 2011; Wysocki et al., 2006). The wider confidence intervals in boat noise and pile driving noise‐off conditions reflect the increased variability of startling, driven by higher occurrences of whole‐school startling events compared to respective noise‐on and control conditions (Figures 7a and 8a). Though a small dataset revealed these trends in the boat noise and pile driving noise‐off conditions, these whole‐school startling events still represent a relatively large proportion of the overall data for those time periods (~4%–20% of startle responses; See Supplemental Information for more details, Tables S17–S22, Figures S14 and S15). There was a decrease in startling behaviour in the boat noise condition over time (Figure 7b), suggesting habituation to boat noise exposure.

Wind farm noise had a different effect, with an increase in startling overall, especially when the noise was on—particularly in the evening. Wind farm noise likely also masked potential predator sounds, but its chronic nature may have resulted in an increase in perceived danger and vigilance, thereby increasing startling especially in the evening in reduced light conditions (van der Knaap, Ashe, et al., 2022). This observation is supported by other species which show increased cortisol levels in response to wind farm noise which is thought to up‐regulate the genes involved in cortisol synthesis, potentially sensitizing the fish to ambient stressors (Wei et al., 2018) which could increase startling as seen in some seabirds (Beale & Monaghan, 2004). Masking can also have similar effects, with noise altering their ability to process sensory information and decreasing the threshold at which they exhibit anti‐predator responses (e.g., increase in startled individuals; Smith et al., 2004; Spiga et al., 2017). Increased startling behaviour in response to wind farm noise could be detrimental to PSL fitness, as it prolongs behaviour that increases vigilance and decreases time spent foraging, which can negatively affect their energetic quality and availability as prey (Spiga et al., 2017). Elevated cortisol levels threaten fish health, physiology, reproductive development and survival and can reduce their ability to avoid predators (Baker et al., 2013; Barton & Iwama, 1991; Mesa, 1994; Wysocki et al., 2006). Anthropogenic noise can also affect biological activities by misleading animals to perceive the noise as a predator's sound and evoke anti‐predator behaviour, whereby they exhibit escape responses and halt foraging (Dominoni et al., 2020).

Only the control treatment showed a significantly higher proportion of startled fish during predator presentations (Figure 6), consistent with the expected anti‐predator behaviour response of teleost fishes to an immediate threat (Kelley & Magurran, 2003). Conversely, predator presentations did not significantly change the proportion of startled fish in any of the noise treatments. One possible explanation is that noise exposure influenced the behavioural state of PSL, resulting in elevated baseline stress levels and vigilance that reduced their response to an additional predation cue. Alternatively, the noise playbacks might have masked or distracted fish from detecting the predator stimulus, thereby diminishing their behavioural response (El‐Dairi et al., 2024). These findings suggest that playbacks of anthropogenic noise can alter routine anti‐predator behaviour, reducing the likelihood of predator avoidance in noisy conditions.

### 4.5 Cloud cover and exposure to noise treatments over time

In general, cloud cover did not strongly influence trends observed in the relative number of fish in the water column and proportion of startled PSL and is likely driven by outliers (i.e., higher proportion of fish in the water column, and entire‐school startle events), though these values were distributed throughout. Nevertheless, the proportion of startled fish remained relatively stable across low to moderate cloud cover but showed a slight decline under highest cloud cover conditions in boat noise (Figure 7c), pile driving (Figure 8b) and wind farm treatments (Figure 9c). Light intensity has been shown to strongly influence behaviour of schooling fish and their ability to acquire visual information from their environment (Xue et al., 2023). As cloud cover increases, prey fish experience reduced visual detection distances, limiting their ability to discern approaching predators or other stimuli (Cerri, 1983; Schmitz & Wainwright, 2011). Poor visual conditions may also impair prey responsiveness by lowering the chances that threatening stimuli are detected (Attwell et al., 2021; Domenici, 2002). These effects may have been further compounded by the noise playbacks. Under high cloud cover conditions, where visual information is already limited, the additional heightened stress levels imposed by noise may have acted to reduce the ability of PSL to detect the stimulus, contributing to a slightly lower proportion of startled fish under high cloud cover. Although the effect of cloud cover on startling and buried fish is influenced by outliers, it suggests that high levels of cloud cover along with playbacks of anthropogenic noise reduce the ability for PSL to detect and respond to information from their environment, such as predatory cues.

Aside from a few trends, we observed slight impacts in the effects of noise exposure over time on the two measured anti‐predator behaviours in PSL. For instance, there is a clear downward trend of the proportion of fish in the water column during boat noise on conditions over time (Figure 7b), while boat noise‐off and control conditions have a slight decrease. Although these trends in the boat noise conditions only span 3 days, this might suggest that PSL bury more over time to conserve energy in response to stressful noise exposure and to avoid further perceived risk. As such, this behaviour might reflect a cumulative behavioural response to repeated disturbance. This decreasing trend in the proportion of fish over time was also shown in other noise treatments, but to a lesser degree (i.e., pile driving noise off; Figure 4b, and wind farm conditions; Figure 5b). These trends, however, were likely driven by an uneven bulk of data points on specific days of exposure. As for startle responses, both boat noise (Figure 7b) and wind farm noise conditions (Figure 9b) showed slight decreases over time, suggesting that PSL have gradually habituated to repeated acoustic exposure. A reduction in this behaviour may allow fish to avoid the energetic costs associated with repeated escape responses to non‐threatening stimuli (Neo et al., 2018). However, habituation can also reduce their ability to detect biologically relevant cues if noise is masking or distracting them from key environmental information, potentially increasing their susceptibility to predation (Simpson et al., 2016). Although we observed modest trends in the proportion of buried and startled fish over time, these results suggest that repeated noise exposure can progressively influence how PSL balance predator avoidance with the energetic costs of responding to repeated disturbance, potentially affecting their ability to perceive and respond to cues in their environment.

### 4.6 Diel patterns in PSL behaviour

In general, the proportion of PSL in the water column peaked in late morning and decreased in the afternoon through the evening. Our results align with similar diel patterns reported in spring and summer in wild PSL (Bizzarro et al., 2016; Hobson, 1986), and other *Ammodytes* species (*A. dubius* and *A. americanus*; Morrison & Davoren, 2024; *A. marinus*; Freeman et al., 2004; Friedlaender et al., 2009; Winslade, 1974; *A. japonicus*; *A. heian*; Amiya et al., 2023; Yamashita et al., 1985), which are likely due to their reliance on sight for foraging, and tendency to bury once they have acquired enough food resources for the day (Morrison & Davoren, 2024; Winslade, 1974; Yamashita et al., 1985). Foraging arena theory (Ahrens et al., 2012; Walters & Juanes, 1993) details the inherent trade‐offs between active foraging and predation risk for most fishes. For PSL, it is thought that as the risk of depredation is high, once enough food is acquired (a critical threshold is reached), the cost of staying in the water column is much higher than any benefit of continued foraging and so they bury (Ahrens et al., 2012; Baker et al., 2019). Although there is evidence that some predators can still access them when buried (e.g., humpback whales; Friedlaender et al., 2009; Hain et al., 1995), sand lance are most commonly preyed on in the water column, where they are more readily accessible to various fish, avian and mammalian predators (van der Kooij et al., 2008). While many studies report diurnal activity for PSL, some studies have shown crepuscular activity during the fall, which coincides with their winter dormancy (Baker et al., 2023; Sisson & Baker, 2017) and suggests that PSL may alter their activity in the water column across the season. One caveat for the wind farm noise acoustic environment is that the experimental tank likely experienced the lowest light levels of all tanks as it was positioned behind the boat noise tank in relation to the windows. Although this difference in light was minor, it could have impacted PSL perception of time of day, resulting in a later emergence in the morning and a earlier cessation of activity in the evening. However, as we did not see any differences from control fish in the morning hours, and all observations began~3.5 h after civil twilight ended (~05:26 on May 19th) and stopped ~1.75 h before civil twilight started (~20:51 on May 19th) we believe that this is unlikely.

### 4.7 Methodological limitations

While we feel that this paper is valuable as the first to examine the behavioural responses of PSL in response to anthropogenic noise, the results of this study need to be taken with caution due to our methodological limitations. This experiment was intended as a pilot to determine how playback type and duration influenced PSL as a key prey source and in turn could be used to inform future noise experiments with a more targeted experimental approach. First, as we were limited in both the number and size of tanks we could use and wanted to examine all types of noise (separate noise in each tank) rather than one (noise and control replicates in two different tanks), each school is a pseudoreplication, limiting our ability to determine between‐sample variation in response to playback stimulus and weakening our statistical inference. Second, as our experiment was cut short, half of our treatments had either too few noise‐off or noise‐on days to be able to examine the effects of cumulative noise effects or rest. Third, as we varied both noise type and playback schedules for each noise treatment to more closely replicate ‘natural conditions’, we cannot separate the effect of sound type and playback schedule (i.e., noise exposure). Fourth, since each playback was in a different tank, and the two sets of tanks differed in both size and relationship to light (i.e. were inside behind windows), comparison of wind farm and boat noise fish to control fish is statistically a bit of a grey zone—as the inside fish may behave differently from control fish due to tank rather than treatment. Finally, as one tank was set back farther from the natural light source compared to the others (wind farm tank) perceived daylight length could have affected burying responses. While these limitations exist in this experiment, we still feel that this experiment offers a useful starting point for other studies examining the short‐ and long‐term effects of different types of noise on important forage fish like PSL and highlights the importance of determining if and what effects noise may have on important fish and their relationships with their predators.

### 4.8 Broader implications and future directions

We found that mid‐term exposure to playbacks of anthropogenic noise can influence PSL behaviour, resulting in changes in fish presence in the water column and startling behaviour. It is likely that playbacks of impulsive and variable noise distracted PSL which could make them more accessible to their predators in the short term, but less accessible in the long term due to depletion (Kok et al., 2023). Chronic noise likely induced stress, which could result in increased burying, making them less accessible to their predators. While all playbacks of noise sources altered behaviour in our experiment, why fish exposed to playbacks of wind farm noise responded so differently to fish exposed to playbacks of other noise sources raises a number of questions. Wind farms also raise a different challenge, as studies have shown that operating wind farms create hard structure allowing for colonization by a number of organisms, often acting as artificial reefs resulting in increased biodiversity (Bicknell et al., 2025; Bicknell et al., 2026). The increase in biodiversity (potential prey), the temporary removal of predators and the alteration of the substrate grain size have all been shown to alter local abundance of Atlantic *Ammodytes* species near operating windfarms (Leonhard et al., 2011; Vandendriessche et al., 2015; van Deurs et al., 2012). While there appears to be an initial increase in Atlantic *Ammodytes* presence at wind farms for some species, numbers tend to return to pre‐wind farm levels or lower in the longer‐term depending on the species. If or how noise generated by operating wind farms interacts with these other factors, impacts Atlantic species or Pacific species remains unknown.

As few studies have directly compared different sound sources, we still know relatively little about how different noise frequency profiles and temporal patterns impact behaviour (Blom et al., 2019; Nichols et al., 2015; Spiga et al., 2017). But, as the marine soundscape is a cacophony of different sounds from different sources, understanding how various types of sounds may impact fish behaviour differently is important to determine the effects of noise on individual species and their communities. Similarly, many studies tend to focus on single species, not taking into account how changes in behaviour of one species may impact the community they live in. To better understand the long‐term community‐wide effects of noise, these relationships need to be taken into account when examining the effects of noise on behaviour of species that impose bottom‐up effects and structure marine food webs (Cury et al., 2000; Pikitch et al., 2012; von Biela et al., 2019). The types of behavioural changes we observed, could lead to change in predator–prey interactions and ultimately the marine food web (Hilborn et al., 2017). Most previous studies focus on short or mid‐term noise exposure, but noise occurs as a chronic pollutant in the environment, so understanding the long‐term effects is imperative (Kok et al., 2023), as different species may respond to chronic noise differently and may be more likely to synthesize or habituate to noise (Blom et al., 2019; Kok et al., 2023). Finally, forage fish availability is strongly dependent on environmental conditions (Field et al., 2009) which could be exacerbated by anthropogenic noise exposure and/or changes in predator–prey relationships (e.g., overexploitation by predators). Identifying the consequences of noise is a critical step towards mitigation and policies designed to minimize noise pollution in marine habitats.

## AUTHOR CONTRIBUTIONS

**Meredith A. V. White**: Conceptualization, Methodology, Investigation, Formal Analysis, Writing—Original Draft, Writing—Review & Editing, Visualization and Funding Acquisition; **Francis Juanes**: Conceptualization, Writing—Review & Editing and Funding Acquisition.; **Jose Tavera**: Investigation and Writing—Review & Editing; **Patrick D. O'Hara**: Conceptualization, Formal Analysis, Writing—Review & Editing and Funding Acquisition; **Matthew R. Baker**: Methodology and Writing—Review & Editing; **Douglas F. Bertram**: Writing—Review & Editing; **Adam Summers**: Methodology and Resources; **David A. Fifield**: Resources and Writing—Review & Editing; **Nora V. Carlson**: Conceptualization, Methodology, Formal Analysis, Resources, Writing—Review & Editing, Visualization, Supervision, Project Administration and Funding Acquisition.

## FUNDING INFORMATION

This project was supported through funding from Mitacs (#IT29508), Environment and Climate Change Canada (GCXE23C381), University of Victoria (Jamie Cassels Undergraduate Research Award) and Natural Sciences and Engineering Research Council (2021‐03288), and Wildlife Conservation Society Canada.

## Supporting information


**Data S1.** Final PSL R Code R1.R: The R code used to run the statistics for the GLMMs found in the results.

**Data S2.** PSL Behav Data R1 May2026.csv: The data used to run all models found in this manuscript.

**Table S1.** Model output for a binomial generalized linear mixed effects model testing effect of all model factors aside from playback type on relative total number of Pacific sand lance (*Ammodytes personatus*) in water column from the *control* condition.
**Table S2.** Type II Wald chi‐square test on binomial generalized linear mixed effects model to test the significant effects of various factors on the relative number of Pacific sand lance (*Ammodytes personatus*) in the water column from the *control* condition.
**Table S3.** Model output for a binomial generalized linear mixed effects model testing effect of *boat noise* and the *control* on relative total number of Pacific sand lance (*Ammodytes personatus*) in water column.
**Table S4.** Type II Wald chi‐square test on binomial generalized linear mixed effects model to test the significant effects of *boat noise* and *control* on the relative number of Pacific sand lance (*Ammodytes personatus*) in the water column.
**Table S5.** Model output for a binomial generalized linear mixed effects model testing the effect of *pile driving* noise and the *control* on relative total number of Pacific sand lance (*Ammodytes personatus*) in water column.
**Table S6.** Type II Wald chi‐square test on binomial generalized linear mixed effects model to test the significant effects of *pile driving* noise and *control* on the relative number of Pacific sand lance (*Ammodytes personatus*) in the water column.
**Table S7.** Model output for a binomial generalized linear mixed effects model testing the effect of *wind farm* noise and the *control* on relative total number of Pacific sand lance (*Ammodytes personatus*) in water column.
**Table S8.** Type II Wald chi‐square test on binomial generalized linear mixed effects model to test the significant effects of *wind farm* noise and *control* on the relative number of Pacific sand lance (*Ammodytes personatus*) in the water column.
**Table S9.** Model output for a binomial generalized linear mixed effects model testing effect of all model factors aside from playback type on the startle response of Pacific sand lance (*Ammodytes personatus*) in water column from the *control* condition.
**Table S10.** Type II Wald chi‐square test on binomial generalized linear mixed effects model to test the significant effects of various factors on the startle response of Pacific sand lance (*Ammodytes personatus*) in the water column from the *control* condition.
**Table S11.** Model output for a binomial generalized linear mixed effects model testing effect of *boat noise* and the *control* on the proportion of Pacific sand lance (*Ammodytes personatus*) in the water column that startled.
**Table S12.** Type II Wald chi‐square test on binomial generalized linear mixed effects model to test the significant effects of *boat noise* and *control* on the proportion of Pacific sand lance (*Ammodytes personatus*) in the water column that startled.
**Table S13.** Model output for a binomial generalized linear mixed effects model testing effect of *pile driving* and the *control* on the proportion of Pacific sand lance (*Ammodytes personatus*) in the water column that startled.
**Table S14.** Type II Wald chi‐square test on binomial generalized linear mixed effects model to test the significant effects of *pile driving* and *control* on the proportion of Pacific sand lance (*Ammodytes personatus*) in the water column that startled.
**Table S15.** Model output for a binomial generalized linear mixed effects model testing effect of *wind farm* and the *control* on the proportion of Pacific sand lance (*Ammodytes personatus*) in the water column that startled.
**Table S16.** Type II Wald chi‐square test on binomial generalized linear mixed effects model to test the significant effects of *wind farm* and *control* on the proportion of Pacific sand lance (*Ammodytes personatus*) in the water column that startled.
**Table S17.** Model output for a binomial generalized linear mixed effects model testing effect of *boat noise* and the *control* on proportion of startled Pacific sand lance (*Ammodytes personatus*) in the water column that startled without any proportions over 50%.
**Table S18.** Type II Wald chi‐square test on binomial generalized linear mixed effects model to test the significant effects of *boat noise* and *control* on the proportion of Pacific sand lance (*Ammodytes personatus*) in the water column that startled without any proportions over 50%.
**Table S19.** Model output for a binomial generalized linear mixed effects model testing effect of *pile driving* and the *control* on proportion of startled Pacific sand lance (*Ammodytes personatus*) without any proportions over 50%.
**Table S20.** Type II Wald chi‐square test on binomial generalized linear mixed effects model to test the significant effects of *pile driving* and *control* on the relative number of Pacific sand lance (*Ammodytes personatus*) in the water column without any proportions over 50%.
**Table S21.** Summary statistics of the startle responses of Pacific sand lance (*Ammodytes personatus*) in response to boat noise.
**Table S22.** Summary statistics of the startle responses of Pacific sand lance (*Ammodytes personatus*) in response to pile driving.
**Figure S1.** Schematic showing where recordings for calibration were taken in the (a) small indoor tank and (b) large outdoor tank. Yellow circles indicate the locations we took recordings to determine loudest and quietest areas, and the degree of shading (red – enclosures with fish, purple – enclosures without fish) indicates the loudest areas (degree of shading not calibrated).
**Figure S2.** Figures showing the (a) spectrogram of the ambient noise in experimental Pacific sand lance (*Ammodytes personatus*) tanks during the control period (i.e. silence) and (b) the Pressure Spectral Density (PSD; generated using PAMGuide; recorded audio sample rate = 48,000, window length = 48,000, window overlap = 50%; playback audio sample rate = 44,100, window length = 44,100, window overlap = 50%).
**Figure S3.** Spectrograms of (a) wind farm noise recorded in the Pacific sand lance (*Ammodytes personatus*) tank and (b) the original recording played back, and PSD plots of (c) wind farm noise recorded in the Pacific sand lance (*Ammodytes personatus*) tank and (d) the original playback (PSD generated using PAMGuide; recorded audio sample rate = 48,000, window length = 48,000, window overlap = 50%; playback audio sample rate = 44,100, window length = 44,100, window overlap = 50%).
**Figure S4.** Spectrograms of (a) pile driving noise recorded in the Pacific sand lance (*Ammodytes personatus*) tank and (b) the original recording played back, and PSD plots of (c) pile driving noise recorded in the Pacific sand lance (*Ammodytes personatus*) tank and (d) the original playback (PSD generated using PAMGuide; recorded audio sample rate = 48,000, window length = 48,000, window overlap = 50%; playback audio sample rate = 44,100, window length = 44,100, window overlap = 50%).
**Figure S5.** Spectrograms of (a) boat noise recorded in the Pacific sand lance (*Ammodytes personatus*) tank both (i) a boat passing by and (ii) background shipping noise, and (b) the original recording played back, both (i) a boat passing by and (ii) background shipping noise, and PSD plots of (c) boat noise recorded in the Pacific sand lance (*Ammodytes personatus*) tank, both (i) a boat passing by and (ii) background shipping noise, and (d) the original playback, both (i) a boat passing by and (ii) background shipping noise (recorded audio sample rate = 48,000, window length = 48,000, window overlap = 50%; playback audio sample rate = 44,100, window length = 44,100, window overlap = 50%).
**Figure S6.** Model validation graphs for a binomial generalized linear mixed effects model testing effect of factors in the control condition on the relative number of Pacific sand lance (*Ammodytes personatus*) in the water column.
**Figure S7.** Model validation graphs for a binomial generalized linear mixed effects model testing effect of the boat noise acoustic environment on the relative number of Pacific sand lance (*Ammodytes personatus*) in the water column.
**Figure S8.** Model validation graphs for a binomial generalized linear mixed effects model testing effect of the pile driving acoustic environment on the relative number of Pacific sand lance (*Ammodytes personatus*) in the water column.
**Figure S9.** Model validation graphs for a binomial generalized linear mixed effects model testing effect of the wind farm acoustic environment on the relative number of Pacific sand lance (*Ammodytes personatus*) in the water column.
**Figure S10.** Model validation graphs for a binomial generalized linear mixed effects model testing effect of factors in the control condition on the proportion of Pacific sand lance (*Ammodytes personatus*) in the water column that startled.
**Figure S11.** Model validation graphs for a binomial generalized linear mixed effects model testing the effect of the boat noise acoustic environment on the proportion of Pacific sand lance (*Ammodytes personatus*) in the water column that startled.
**Figure S12.** Model validation graphs diagnostics for a binomial generalized linear mixed effects model testing the effect of the pile driving acoustic environment on the proportion of Pacific sand lance (*Ammodytes personatus*) in the water column that startled.
**Figure S13.** Model validation graphs for a binomial generalized linear mixed effects model testing the effect of the wind farm noise acoustic environment on the proportion of Pacific sand lance (*Ammodytes personatus*) in the water column that startled.
**Figure S14.** Histogram showing the spread of startling data for control, boat noise‐on and boat noise‐off data.
**Figure S15.** Histogram showing the spread of startling data for control, pile driving on and pile driving off data.

## Data Availability

All data and R code used in this paper are available in the SI (Final PSL R Code R1.R & PSL Behav Data R1 May2026.csv).
