# Supplementary material for: The effects of playbacks of anthropogenic noise on the anti‐predator behaviour of Pacific sand lance, Ammodytes personatus
Source: J Fish Biol. 2026 Sep 7;109(5):2669–91. doi: 10.1111/jfb.70642 (PMC13770698; doi:10.1111/jfb.70642)
Supplement: Supplementary file 1 [file JFB-109-2669-s001.docx]

**Supplemental Information**

**Table S1.** Model output for a binomial generalized linear mixed effects model testing effect of all model factors aside from playback type on relative total number of Pacific sand lance (*Ammodytes personatus*) in water column from the *control* condition.

Model: glmer(cbind(PoolNo-TotalNumber,TotalNumber)~exposure + time+ predator + feeding+ cloudcover + (1|EnclosureID)+ (1| Day), data= subset(onetime, Stimulus == "Control"), family=binomial(link="logit"))

| Random effects: |  |  |  |  |
| --- | --- | --- | --- | --- |
|  | Groups | Name | Variance | Std.Dev. |
|  | Day | (Intercept) | 0.03 | 0.16 |
|  | Enclosure | (Intercept) | 0.11 | 0.34 |
| Number of obs: 132, groups: Day, 11; Enclosure, 2 | | | | |
|  |  |  |  |  |
| Fixed effects: |  |  |  |  |
|  | Estimate | Std.Error | z | p |
| (Intercept) | 0.17 | 0.28 | 0.62 | 0.537 |
| time Afternoon | 0.26 | 0.06 | 4.31 | < 0.001 |
| time Evening | 0.53 | 0.06 | 8.91 | < 0.001 |
| exposure | 0.01 | 0.02 | 0.64 | 0.522 |
| predator y | 0.13 | 0.13 | 1.01 | 0.310 |
| feeding y | -0.05 | 0.11 | -0.45 | 0.652 |
| cloud_cover | -0.11 | 0.11 | -0.99 | 0.322 |

**Table S2.** Type II Wald chi-square test on binomial generalized linear mixed effects model to test the significant effects of various factors on the relative number of Pacific sand lance (*Ammodytes personatus*) in the water column from the *control* condition

|  | Chisq | Df | P |
| --- | --- | --- | --- |
| time | 79.43 | 2 | **< 0.001** |
| exposure | 0.41 | 1 | 0.522 |
| predator | 1.03 | 1 | 0.311 |
| feeding | 0.20 | 1 | 0.652 |
| cloud_cover | 0.98 | 1 | 0.322 |

**Table S3.** Model output for a binomial generalized linear mixed effects model testing effect of *boat noise* and the *control* on relative total number of Pacific sand lance (*Ammodytes personatus*) in water column.

Model: glmer(cbind(PoolNo-TotalNumber,TotalNumber)~playback_state*exposure + playback_state*time+ predator + feeding+ cloud cover + (1|EnclosureID)+ (1| Day), data= subset(onetime, Stimulus == "Boat Noise"| Stimulus == "Control"), family=binomial(link="logit"))

| Random effects: |  |  |  |  |
| --- | --- | --- | --- | --- |
|  | Groups | Name | Variance | Std.Dev. |
|  | Day | (Intercept) | 0.03 | 0.17 |
|  | Enclosure | (Intercept) | 0.05 | 0.23 |
| Number of obs: 264, groups: Day, 11; Enclosure, 4 | | | | |
|  |  |  |  |  |
| Fixed effects: |  |  |  |  |
|  | Estimate | Std.Error | Z | P |
| (Intercept) | 0.22 | 0.20 | 1.10 | 0.270 |
| time Afternoon | 0.28 | 0.06 | 4.64 | < 0.001 |
| time Evening | 0.53 | 0.06 | 8.94 | < 0.001 |
| playback Off | -0.23 | 0.27 | -0.86 | 0.393 |
| playback On | 0.31 | 0.28 | 1.12 | 0.262 |
| exposure | 0.01 | 0.01 | 0.96 | 0.335 |
| predator y | 0.09 | 0.10 | 0.90 | 0.371 |
| feeding y | -0.02 | 0.11 | -0.20 | 0.846 |
| cloud_cover | -0.24 | 0.09 | -2.71 | 0.007 |
| playback Off : time Afternoon | -0.18 | 0.11 | -1.72 | 0.086 |
| playback Off : time Evening | 0.27 | 0.11 | 2.41 | 0.016 |
| playback On : time Afternoon | -0.42 | 0.11 | -3.90 | 0.000 |
| playback On : time Evening | 0.26 | 0.11 | 2.35 | 0.019 |
| playback Off: exposure | 0.19 | 0.07 | 2.65 | 0.008 |
| playback On: exposure | -0.34 | 0.07 | -5.20 | < 0.001 |

**Table S4.** Type II Wald chi-square test on binomial generalized linear mixed effects model to test the significant effects of *boat noise* and *control* on the relative number of Pacific sand lance (*Ammodytes personatus*) in the water column

|  | Chisq | Df | P |
| --- | --- | --- | --- |
| time | 235.71 | 2 | **< 0.001** |
| playback | 18.13 | 2 | **< 0.001** |
| exposure | 0.05 | 1 | 0.831 |
| predator | 0.80 | 1 | 0.371 |
| feeding | 0.04 | 1 | 0.846 |
| cloud_cover | 7.33 | 1 | **0.007** |
| playback:time | 44.83 | 4 | **< 0.001** |
| playback:exposure | 34.21 | 2 | **< 0.001** |

**Table S5.** Model output for a binomial generalized linear mixed effects model testing the effect of *pile driving* noise and the *control* on relative total number of Pacific sand lance (*Ammodytes personatus*) in water column.

Model: glmer(cbind(PoolNo-TotalNumber,TotalNumber)~playback_state*time+exposure+predator + feeding+ cloud cover + (1|EnclosureID)+ (1| Day),data= subset(onetime, Stimulus == "Pile Driving"| Stimulus == "Control"), family=binomial(link="logit"))

| Random effects: |  |  |  |  |
| --- | --- | --- | --- | --- |
|  | Groups | Name | Variance | Std.Dev. |
|  | Day | (Intercept) | 0.05 | 0.23 |
|  | Enclosure | (Intercept) | 0.06 | 0.24 |
| Number of obs: 248, groups: Day, 11; Enclosure, 4 | | | | |
|  |  |  |  |  |
| Fixed effects: |  |  |  |  |
|  | Estimate | Std.Error | Z | P |
| (Intercept) | 0.35 | 0.22 | 1.59 | 0.111 |
| time Afternoon | 0.25 | 0.06 | 4.17 | <0.001 |
| time Evening | 0.53 | 0.06 | 8.90 | <0.001 |
| playback Off | -0.37 | 0.26 | -1.44 | 0.149 |
| playback On | -0.23 | 0.27 | -0.85 | 0.394 |
| exposure | -0.03 | 0.01 | -2.77 | 0.006 |
| predator y | 0.18 | 0.11 | 1.60 | 0.111 |
| feeding y | -0.07 | 0.14 | -0.48 | 0.630 |
| cloud_cover | -0.03 | 0.09 | -0.29 | 0.772 |
| playback Off : time Afternoon | -0.32 | 0.10 | -3.32 | 0.001 |
| playback Off : time Evening | 0.01 | 0.10 | 0.10 | 0.920 |
| playback On : time Afternoon | -0.27 | 0.14 | -1.93 | 0.054 |
| playback On : time Evening | -0.02 | 0.14 | -0.12 | 0.906 |

**Table S6.** Type II Wald chi-square test on binomial generalized linear mixed effects model to test the significant effects of *pile driving* noise and *control* on the relative number of Pacific sand lance (*Ammodytes personatus*) in the water column

|  | Chisq | Df | P |
| --- | --- | --- | --- |
| time | 152.33 | 2 | **<0.001** |
| playback | 6.32 | 2 | **0.043** |
| exposure | 7.69 | 1 | **0.006** |
| predator | 2.54 | 1 | 0.111 |
| feeding | 0.23 | 1 | 0.630 |
| cloud_cover | 0.08 | 1 | 0.772 |
| playback:time | 16.13 | 4 | **0.003** |

**Table S7.** Model output for a binomial generalized linear mixed effects model testing the effect of *wind farm* noise and the *control* on relative total number of Pacific sand lance (*Ammodytes personatus*) in water column.

Model: glmer(cbind(PoolNo-TotalNumber,TotalNumber)~time*playback_state+exposure+ predator+ feeding+ cloud cover + (1|EnclosureID)+ (1| Day) ,data= subset(onetime, Stimulus == "Wind Farm" | Stimulus == "Control"), family=binomial(link="logit"))

| Random effects: |  |  |  |  |
| --- | --- | --- | --- | --- |
|  | Groups | Name | Variance | Std.Dev. |
|  | Day | (Intercept) | 0.56 | 0.75 |
|  | Enclosure | (Intercept) | 0.11 | 0.33 |
| Number of obs: 257, groups: Day, 11; Enclosure, 4 | | | | |
|  |  |  |  |  |
| Fixed effects: |  |  |  |  |
|  | Estimate | Std.Error | Z | P |
| (Intercept) | -0.17 | 0.36 | -0.49 | 0.626 |
| time Afternoon | 0.27 | 0.06 | 4.46 | < 0.001 |
| time Evening | 0.54 | 0.06 | 8.94 | < 0.001 |
| playback Off | 0.58 | 0.36 | 1.60 | 0.110 |
| playback On | 0.54 | 0.34 | 1.58 | 0.115 |
| exposure | 0.17 | 0.01 | 13.12 | < 0.001 |
| predator y | 0.47 | 0.50 | 0.94 | 0.346 |
| feeding y | -1.21 | 0.15 | -8.26 | < 0.001 |
| cloud_cover | -0.17 | 0.11 | -1.60 | 0.110 |
| playback Off : time Afternoon | 0.57 | 0.19 | 3.03 | 0.002 |
| playback Off : time Evening | 1.84 | 0.28 | 6.46 | < 0.001 |
| playback On : time Afternoon | 0.45 | 0.10 | 4.66 | < 0.001 |
| playback On : time Evening | 2.14 | 0.13 | 16.19 | < 0.001 |

**Table S8.** Type II Wald chi-square test on binomial generalized linear mixed effects model to test the significant effects of *wind farm* noise and *control* on the relative number of Pacific sand lance (*Ammodytes personatus*) in the water column

|  | Chisq | Df | P |
| --- | --- | --- | --- |
| time | 393.07 | 2 | **< 0.001** |
| playback | 9.07 | 2 | **0.011** |
| exposure | 172.25 | 1 | **< 0.001** |
| predator | 0.89 | 1 | 0.346 |
| feeding | 68.21 | 1 | **< 0.001** |
| cloud_cover | 2.56 | 1 | 0.110 |
| playback:time | 288.04 | 4 | **< 0.001** |

**Table S9.** Model output for a binomial generalized linear mixed effects model testing effect of all model factors aside from playback type on the startle response of Pacific sand lance (*Ammodytes personatus*) in water column from the *control* condition.

Model: glmer(cbind(TotalNumber-StartleAdjusted,StartleAdjusted)~exposure + time+ predator + feeding+ cloudcover + (1|EnclosureID)+ (1| Day), data= subset(onetime, Stimulus == "Control"), family=binomial(link="logit"))

| Random effects: |  |  |  |  |
| --- | --- | --- | --- | --- |
|  | Groups | Name | Variance | Std.Dev. |
|  | Day | (Intercept) | 0.28 | 0.53 |
|  | Enclosure | (Intercept) | 0.02 | 0.15 |
| Number of obs: 132, groups: Day, 11; Enclosure, 2 | | | | |
|  |  |  |  |  |
| Fixed effects: |  |  |  |  |
|  | Estimate | Std.Error | z | p |
| (Intercept) | 3.59 | 0.54 | 6.61 | < 0.001 |
| time Afternoon | -0.22 | 0.26 | -0.85 | 0.397 |
| time Evening | -0.13 | 0.27 | -0.47 | 0.641 |
| exposure | 0.06 | 0.06 | 0.97 | 0.330 |
| predator y | -0.98 | 0.45 | -2.19 | 0.029 |
| feeding y | 0.39 | 0.40 | 0.98 | 0.327 |
| cloud_cover | -0.31 | 0.48 | -0.65 | 0.519 |

**Table S10.** Type II Wald chi-square test on binomial generalized linear mixed effects model to test the significant effects of various factors on the startle response of Pacific sand lance (*Ammodytes personatus*) in the water column from the *control* condition

|  | Chisq | Df | P |
| --- | --- | --- | --- |
| time | 0.72 | 2 | 0.697 |
| exposure | 0.95 | 1 | 0.330 |
| predator | 4.79 | 1 | **0.029** |
| feeding | 0.96 | 1 | 0.326 |
| cloud_cover | 0.42 | 1 | 0.519 |

**Table S11**. Model output for a binomial generalized linear mixed effects model testing effect of *boat noise* and the *control* on the proportion of Pacific sand lance (*Ammodytes personatus*) in the water column that startled.

Model: glmer(cbind(TotalNumber-StartleAdjusted,StartleAdjusted)~playback_state*time + playback_state*exposure +predator + feeding+ cloud cover + (1|EnclosureID)+ (1| Day), data= subset(onetime, Stimulus == "Boat Noise"| Stimulus == "Silence"), family=binomial(link="logit"),glmerControl(optimizer ='optimx', optCtrl=list(method='nlminb')))

| Random effects: |  |  |  |  |
| --- | --- | --- | --- | --- |
|  | Groups | Name | Variance | Std.Dev. |
|  | Day | (Intercept) | 0.68 | 0.82 |
|  | Enclosure | (Intercept) | 0.22 | 0.47 |
| Number of obs: 264, groups: Day, 11; Enclosure, 4 | | | | |
|  |  |  |  |  |
| Fixed effects: |  |  |  |  |
|  | Estimate | Std.Error | Z | P |
| (Intercept) | 4.65 | 0.64 | 7.32 | < 0.001 |
| time Afternoon | -0.19 | 0.25 | -0.75 | 0.455 |
| time Evening | -0.08 | 0.27 | -0.31 | 0.758 |
| playback Off | -0.11 | 0.59 | -0.18 | 0.854 |
| playback On | -1.65 | 0.76 | -2.18 | 0.029 |
| exposure | -0.05 | 0.05 | -1.10 | 0.273 |
| predator y | -0.88 | 0.48 | -1.84 | 0.066 |
| feeding y | 0.30 | 0.52 | 0.58 | 0.561 |
| cloud_cover | -1.12 | 0.38 | -2.93 | 0.003 |
| playback Off : time Afternoon | -0.80 | 0.33 | -2.43 | 0.015 |
| playback Off : time Evening | 0.63 | 0.41 | 1.54 | 0.124 |
| playback On : time Afternoon | 0.91 | 0.43 | 2.14 | 0.033 |
| playback On : time Evening | 1.15 | 0.53 | 2.17 | 0.030 |
| playback Off: exposure | -0.90 | 0.25 | -3.54 | < 0.001 |
| playback On: exposure | 0.54 | 0.30 | 1.82 | 0.069 |

**Table S12.** Type II Wald chi-square test on binomial generalized linear mixed effects model to test the significant effects of *boat noise* and *control* on the proportion of Pacific sand lance (*Ammodytes personatus*) in the water column that startled.

|  | Chisq | Df | P |
| --- | --- | --- | --- |
| time | 19.36 | 2 | **< 0.001** |
| playback | 15.18 | 2 | **0.001** |
| exposure | 0.21 | 1 | 0.649 |
| predator | 3.38 | 1 | 0.066 |
| feeding | 0.34 | 1 | 0.561 |
| cloud_cover | 8.55 | 1 | **0.003** |
| playback:time | 29.77 | 4 | **< 0.001** |
| playback:exposure | 15.86 | 2 | **< 0.001** |

**Table S13.** Model output for a binomial generalized linear mixed effects model testing effect of *pile driving* and the *control* on the proportion of Pacific sand lance (*Ammodytes personatus*) in the water column that startled.

Model: glmer(cbind(TotalNumber-StartleAdjusted,StartleAdjusted)~playback_state*time+ exposure+ predator+ feeding+(1|EnclosureID)+ (1| Day),data= subset(onetime, Stimulus == "Pile Driving"| Stimulus == "Silence"), family=binomial(link="logit"))

| Random effects: |  |  |  |  |
| --- | --- | --- | --- | --- |
|  | Groups | Name | Variance | Std.Dev. |
|  | Day | (Intercept) | 0.74 | 0.86 |
|  | Enclosure | (Intercept) | <0.01 | 0.01 |
| Number of obs: 257, groups: Day, 11; Enclosure, 4 | | | | |
|  |  |  |  |  |
| Fixed effects: |  |  |  |  |
|  | Estimate | Std.Error | Z | P |
| (Intercept) | 4.58 | 0.56 | 8.15 | <0.001 |
| time Afternoon | -0.16 | 0.25 | -0.63 | 0.531 |
| time Evening | -0.13 | 0.27 | -0.49 | 0.626 |
| playback Off | -0.65 | 0.30 | -2.15 | 0.032 |
| playback On | -0.53 | 0.50 | -1.05 | 0.292 |
| exposure | 0.01 | 0.05 | 0.21 | 0.836 |
| predator y | -0.72 | 0.50 | -1.43 | 0.153 |
| feeding y | 0.06 | 0.53 | 0.11 | 0.914 |
| cloud_cover | -1.28 | 0.40 | -3.19 | 0.001 |
| playback Off : time Afternoon | -0.72 | 0.32 | -2.25 | 0.025 |
| playback Off : time Evening | 0.30 | 0.38 | 0.79 | 0.432 |
| playback On : time Afternoon | 1.62 | 0.73 | 2.22 | 0.026 |
| playback On : time Evening | 0.59 | 0.63 | 0.94 | 0.350 |

**Table S14.** Type II Wald chi-square test on binomial generalized linear mixed effects model to test the significant effects of *pile driving* and *control* on the proportion of Pacific sand lance (*Ammodytes personatus*) in the water column that startled.

|  | Chisq | Df | P |
| --- | --- | --- | --- |
| time | 14.56 | 2 | **0.001** |
| playback | 18.92 | 2 | **<0.001** |
| exposure | 0.04 | 1 | 0.836 |
| predator | 2.04 | 1 | 0.153 |
| feeding | 0.01 | 1 | 0.914 |
| cloud_cover | 10.20 | 1 | **0.001** |
| playback:time | 18.96 | 4 | **0.001** |

**Table S15.** Model output for a binomial generalized linear mixed effects model testing effect of *wind farm* and the *control* on the proportion of Pacific sand lance (*Ammodytes personatus*) in the water column that startled.

Model: glmer(cbind(TotalNumber-StartleAdjusted,StartleAdjusted)~playback_state*time+exposure+predator+ feeding+(1|EnclosureID)+(1| Day),data= winddata, family=binomial(link="logit"))

| Random effects: |  |  |  |  |
| --- | --- | --- | --- | --- |
|  | Groups | Name | Variance | Std.Dev. |
|  | Day | (Intercept) | 1.22 | 1.10 |
|  | Enclosure | (Intercept) | 0.11 | 0.33 |
| Number of obs: 257, groups: Day, 11; Enclosure, 4 | | | | |
|  |  |  |  |  |
| Fixed effects: |  |  |  |  |
|  | Estimate | Std.Error | Z | P |
| (Intercept) | 5.44 | 0.61 | 8.97 | < 0.001 |
| time Afternoon | -0.12 | 0.26 | -0.46 | 0.644 |
| time Evening | -0.04 | 0.27 | -0.13 | 0.894 |
| playback Off | 0.12 | 0.64 | 0.20 | 0.845 |
| playback On | -2.43 | 0.42 | -5.75 | 0.000 |
| exposure | -0.17 | 0.05 | -3.33 | 0.001 |
| predator y | -1.22 | 0.78 | -1.58 | 0.115 |
| feeding y | 0.15 | 0.36 | 0.42 | 0.674 |
| cloud_cover | -1.00 | 0.39 | -2.56 | 0.010 |
| playback Off : time Afternoon | -1.36 | 0.62 | -2.20 | 0.028 |
| playback Off : time Evening | -1.03 | 1.17 | -0.88 | 0.378 |
| playback On : time Afternoon | 1.22 | 0.34 | 3.54 | 0.000 |
| playback On : time Evening | -0.64 | 0.40 | -1.59 | 0.112 |

**Table S16.** Type II Wald chi-square test on binomial generalized linear mixed effects model to test the significant effects of *wind farm* and *control* on the proportion of Pacific sand lance (*Ammodytes personatus*) in the water column that startled.

|  | Chisq | Df | P |
| --- | --- | --- | --- |
| time | 5.27 | 2 | **0.072** |
| playback | 38.11 | 2 | **< 0.001** |
| exposure | 11.06 | 1 | **0.001** |
| predator | 2.49 | 1 | 0.115 |
| feeding | 0.18 | 1 | 0.674 |
| cloud_cover | 6.56 | 1 | **0.010** |
| playback:time | 31.52 | 4 | **< 0.001** |

**Table S17**. Model output for a binomial generalized linear mixed effects model testing effect of *boat noise* and the *control* on proportion of startled Pacific sand lance (*Ammodytes personatus*) in the water column that startled without any proportions over 50%.

Model: glmer(cbind(TotalNumber-StartleAdjusted,StartleAdjusted)~playback_state*time + playback_state*exposure +predator + feeding+ cloud cover + (1|EnclosureID)+ (1| Day), data= subset(boatno, propstartle<.50), family=binomial(link="logit"),glmerControl(optimizer ='optimx', optCtrl=list(method='nlminb')))

| Random effects: |  |  |  |  |
| --- | --- | --- | --- | --- |
|  | Groups | Name | Variance | Std.Dev. |
|  | Day | (Intercept) | 0.33 | 0.57 |
|  | Enclosure | (Intercept) | 0.02 | 0.13 |
| Number of obs: 264, groups: Day, 11; Enclosure, 4 | | | | |
|  |  |  |  |  |
| Fixed effects: |  |  |  |  |
|  | Estimate | Std.Error | Z | P |
| (Intercept) | 4.26 | 0.49 | 8.75 | <2e-16 |
| playback Off | -0.53 | 0.44 | -1.18 | 0.237 |
| playback On | -1.21 | 0.60 | -2.01 | 0.045 |
| exposure | -0.07 | 0.05 | -1.40 | 0.161 |
| time Afternoon | -0.21 | 0.26 | -0.83 | 0.405 |
| time Evening | 0.15 | 0.29 | 0.51 | 0.614 |
| predator y | -0.45 | 0.40 | -1.12 | 0.263 |
| feeding y | 0.03 | 0.39 | 0.08 | 0.937 |
| cloud_cover | -0.36 | 0.39 | -0.94 | 0.347 |
| playback Off: exposure | 0.13 | 0.31 | 0.41 | 0.684 |
| playback On: exposure | 0.48 | 0.29 | 1.65 | 0.099 |
| playback Off : time Afternoon | -0.02 | 0.42 | -0.05 | 0.960 |
| playback On : time Afternoon | 0.30 | 0.44 | 0.67 | 0.506 |
| playback Off : time Evening | 0.07 | 0.52 | 0.14 | 0.886 |
| playback On : time Evening | 0.29 | 0.56 | 0.53 | 0.597 |

**Table S18.** Type II Wald chi-square test on binomial generalized linear mixed effects model to test the significant effects of *boat noise* and *control* on the proportion of Pacific sand lance (*Ammodytes personatus*) in the water column that startled without any proportions over 50%.

|  | Chisq | Df | P |
| --- | --- | --- | --- |
| playback | 2.04 | 2 | 0.361 |
| time | 3.16 | 2 | 0.206 |
| exposure | 2.05 | 1 | 0.152 |
| predator | 1.25 | 1 | 0.263 |
| feeding | 0.01 | 1 | 0.937 |
| cloud_cover | 0.88 | 1 | 0.347 |
| playback:time | 0.61 | 4 | 0.961 |
| playback:exposure | 2.89 | 2 | 0.236 |

**Table S19.** Model output for a binomial generalized linear mixed effects model testing effect of *pile driving* and the *control* on proportion of startled Pacific sand lance (*Ammodytes personatus*) without any proportions over 50%.

Model: glmer(cbind(TotalNumber-StartleAdjusted,StartleAdjusted)~playback_state*time+ exposure+ predator+ feeding+(1|EnclosureID)+ (1| Day),data= subset(pileno, propstartle<.50), family=binomial(link="logit"))

| Random effects: |  |  |  |  |
| --- | --- | --- | --- | --- |
| Groups | Name | Variance | Std.Dev. |  |
| Day | (Intercept) | 0.18 | 0.42 |  |
| CameraNumber | (Intercept) | 0.07 | 0.26 |  |
| Number of obs: 266, groups: Day, 12; CameraNumber, 4 | | | |  |
|  |  |  |  |  |
| Fixed effects: |  |  |  |  |
|  | Estimate | Std. Error | z value | Pr(>\|z\|) |
| (Intercept) | 4.00 | 0.51 | 7.87 | 0.000 |
| playback Off | -0.70 | 0.41 | -1.73 | 0.084 |
| playback On | -0.28 | 0.56 | -0.50 | 0.618 |
| exposure | -0.02 | 0.05 | -0.44 | 0.660 |
| time Afternoon | -0.22 | 0.25 | -0.87 | 0.385 |
| time Evening | 0.15 | 0.29 | 0.54 | 0.593 |
| predator y | -0.39 | 0.32 | -1.22 | 0.222 |
| feeding y | -0.07 | 0.30 | -0.22 | 0.824 |
| cloud_cover | -0.32 | 0.38 | -0.86 | 0.392 |
| playback Off : time Afternoon | 0.89 | 0.37 | 2.39 | 0.017 |
| playback On : time Afternoon | 1.34 | 0.73 | 1.84 | 0.066 |
| playback Off : time Evening | 0.41 | 0.42 | 0.98 | 0.329 |
| playback On : time Evening | -0.08 | 0.64 | -0.12 | 0.904 |

**Table S20.** Type II Wald chi-square test on binomial generalized linear mixed effects model to test the significant effects of *pile driving* and *control* on the relative number of Pacific sand lance (*Ammodytes personatus*) in the water column without any proportions over 50%.

|  | Chisq | Df | P |
| --- | --- | --- | --- |
| playback | 1.39 | 2 | 0.500 |
| time | 3.10 | 2 | 0.212 |
| exposure | 0.19 | 1 | 0.660 |
| predator | 1.49 | 1 | 0.222 |
| feeding | 0.05 | 1 | 0.824 |
| cloud_cover | 0.73 | 1 | 0.392 |
| playback:time | 8.65 | 4 | 0.070 |

**Table S21.** Summary statistics of the startle responses of Pacific sand lance (*Ammodytes personatus*) in response to boat noise.

| Stimulus | Zero startle responses | Startle response > 0 | Startle response > 0.05 | Total | Prop all trials > 0.05 | Prop all startles > 0.05 |
| --- | --- | --- | --- | --- | --- | --- |
| Boat noise On | 46 | 26 | 1 | 72 | 0.0139 | 0.0385 |
| Boat noise Off | 37 | 35 | 7 | 72 | 0.0972 | 0.2000 |

**Table S22.** Summary statistics of the startle responses of Pacific sand lance (*Ammodytes personatus*) in response to pile driving.

| Stimulus | Zero startle responses | Startle response > 0 | Startle response > 0.05 | Total | Prop all trials > 0.05 | Prop all startles > 0.05 |
| --- | --- | --- | --- | --- | --- | --- |
| Boat noise On | 26 | 10 | 0 | 36 | 0.0000 | 0.0000 |
| Boat noise Off | 51 | 41 | 5 | 92 | 0.0544 | 0.1220 |

**Figure S1**. Schematic showing where recordings for calibration were taken in the a) small indoor tank, and b) large outdoor tank. Yellow circles indicate the locations we took recordings to determine loudest and quietest areas, and the degree of shading (red – enclosures with fish, purple – enclosures without fish) indicates the loudest areas (degree of shading not calibrated).

**Figure S2.** Figures showing the a) spectrogram of the ambient noise in experimental Pacific sand lance (*Ammodytes personatus*) tanks during the control period (i.e. silence), and b) the Pressure Spectral Density (PSD; generated using PAMGuide; recorded audio sample rate = 48000, window length = 48000, window overlap = 50%; playback audio sample rate = 44100, window length = 44100, window overlap = 50%).

**Figure S3.** Spectrograms of a) wind farm noise recorded in the Pacific sand lance (*Ammodytes personatus*) tank, and b) the original recording played back and PSD plots of c) wind farm noise recorded in the Pacific sand lance (*Ammodytes personatus*) tank, and d) the original playback (PSD generated using PAMGuide; recorded audio sample rate = 48000, window length = 48000, window overlap = 50%; playback audio sample rate = 44100, window length = 44100, window overlap = 50%).

**Figure S4.** Spectrograms of a) pile driving noise recorded in the Pacific sand lance (*Ammodytes personatus*) tank, and b) the original recording played back and PSD plots of c) pile driving noise recorded in the Pacific sand lance (*Ammodytes personatus*) tank, and d) the original playback (PSD generated using PAMGuide; recorded audio sample rate = 48000, window length = 48000, window overlap = 50%; playback audio sample rate = 44100, window length = 44100, window overlap = 50%).

**Figure S5**. Spectrograms of a) boat noise recorded in the Pacific sand lance (*Ammodytes personatus*) tank both i) a boat passing by, and ii) background shipping noise, and b) the original recording played back, both i) a boat passing by, and ii) background shipping noise, and PSD plots of c) boat noise recorded in the Pacific sand lance (*Ammodytes personatus*) tank, both i) a boat passing by, and ii) background shipping noise, and d) the original playback, both i) a boat passing by, and ii) background shipping noise (recorded audio sample rate = 48000, window length = 48000, window overlap = 50%; playback audio sample rate = 44100, window length = 44100, window overlap = 50%).

**Figure S6.** Model validation graphs for a binomial generalized linear mixed effects model testing effect of factors in the control condition on the relative number of Pacific sand lance (*Ammodytes personatus*) in the water column.

Model: glmer(cbind(PoolNo-TotalNumber,TotalNumber)~mornoraft+exposure+ predatorday + daysfed+ CloudcoverCop+(1|CameraNumber)+ (1| Day) ,data= subset(BoatBehav, Stimulus == "Control"), family=binomial(link="logit"))

**Figure S7.** Model validation graphs for a binomial generalized linear mixed effects model testing effect of the boat noise acoustic environment on the relative number of Pacific sand lance (*Ammodytes personatus*) in the water column.

Model: glmer(cbind(PoolNo-TotalNumber,TotalNumber)~ mornoraft*playbackonoff + exposure*playbackonoff + predatorday + daysfed+ CloudcoverCop + (1|CameraNumber)+(1| Day),data= subset(BoatBehav, Stimulus == "Boat Noise"| Stimulus == "Control"), family= binomial(link="logit"), glmerControl(optimizer ='Nelder_Mead', calc.derivs =FALSE ))

**Figure S8.** Model validation graphs for a binomial generalized linear mixed effects model testing effect of the pile driving acoustic environment on the relative number of Pacific sand lance (*Ammodytes personatus*) in the water column.

Model: glmer(cbind(PoolNo-TotalNumber,TotalNumber)~mornoraft*playbackonoff+exposure+ predatorday + daysfed+ +CloudcoverCop+(1|CameraNumber)+ (1| Day),data= subset(BoatBehav, Stimulus == "Pile Driving"| Stimulus == "Control"), family=binomial(link="logit"), glmerControl(optimizer ='Nelder_Mead', calc.derivs =FALSE ))

**Figure S9.** Model validation graphs for a binomial generalized linear mixed effects model testing effect of the wind farm acoustic environment on the relative number of Pacific sand lance (*Ammodytes personatus*) in the water column.

Model: glmer(cbind(PoolNo-TotalNumber,TotalNumber)~mornoraft*playbackonoff+exposure+ predatorday + daysfed+ CloudcoverCop+(1|CameraNumber)+ (1| Day) ,data= subset(BoatBehav, Stimulus == "Wind Farm" | Stimulus == "Control"), family=binomial(link="logit"))

**Figure S10.** Model validation graphs for a binomial generalized linear mixed effects model testing effect of factors in the control condition on the proportion of Pacific sand lance (*Ammodytes personatus*) in the water column that startled.

Model: glmer(cbind(TotalNumber-StartleAdjusted,StartleAdjusted)~mornoraft+exposure+ predatorday + daysfed+ CloudcoverCop+(1|CameraNumber)+ (1| Day) ,data= subset(BoatBehav, Stimulus == "Control"), family=binomial(link="logit"), glmerControl(optimizer ='Nelder_Mead', calc.derivs =FALSE ))

**Figure S11**. Model validation graphs for a binomial generalized linear mixed effects model testing the effect of the boat noise acoustic environment on the proportion of Pacific sand lance (*Ammodytes personatus*) in the water column that startled.

Model: glmer(cbind(TotalNumber-StartleAdjusted,StartleAdjusted) ~mornoraft*playbackonoff +exposure*playbackonoff+ predatorday + daysfed+ +CloudcoverCop+ (1|CameraNumber)+ (1| Day), data= subset(BoatBehav, Stimulus == "Boat Noise"| Stimulus == "Control"), family=binomial(link="logit"), glmerControl(optimizer ='Nelder_Mead', calc.derivs =FALSE ))

**Figure S12.** Model validation graphs diagnostics for a binomial generalized linear mixed effects model testing the effect of the pile driving acoustic environment on the proportion of Pacific sand lance (*Ammodytes personatus*) in the water column that startled.

Model: glmer(cbind(TotalNumber-StartleAdjusted,StartleAdjusted) ~mornoraft*playbackonoff +exposure+ predatorday + daysfed+ CloudcoverCop+ (1|CameraNumber)+ (1| Day), data= subset(BoatBehav, Stimulus == "Pile Driving"| Stimulus == "Control"), family=binomial(link="logit"))

**Figure S13.** Model validation graphs for a binomial generalized linear mixed effects model testing the effect of the wind farm noise acoustic environment on the proportion of Pacific sand lance (*Ammodytes personatus*) in the water column that startled.

Model: glmer(cbind(TotalNumber-StartleAdjusted,StartleAdjusted) ~ mornoraft*playbackonoff +exposure+ predatorday + daysfed+ CloudcoverCop+ (1|CameraNumber)+(1| Day), data= subset(BoatBehav, Stimulus == "Wind Farm" | Stimulus == "Control"), family=binomial(link="logit"))

**Figure S14.** Histogram showing the spread of startling data for control, boat noise on, and boat noise off data.

**Figure S15.** Histogram showing the spread of startling data for control, pile driving on, and pile driving off data.

**Final PSL R Code R1.R:** The R code used to run the statistics for the GLMMs found in the results.

**PSL Behav Data R1 May2026.csv:** The data used to run all models found in this manuscript.
